# WISP1 drives esophageal squamous cell carcinoma progression via modulation of cancer-associated fibroblasts and immune microenvironment

**DOI:** 10.3389/fimmu.2025.1586790

**Published:** 2025-07-23

**Authors:** Zhiqiang Yi, Qiqi Zhang, Ting Tao, Xiaojia Liu, Hui Li, Xiujuan Li, Zhiqiang Zhang

**Affiliations:** ^1^ The Second Department of Gastroenterology, The First Affiliated Hospital of Xinjiang Medical University, State Key Laboratory of Pathogenesis, Prevention and Treatment of High Incidence Diseases in Central Asia, Urumqi, China; ^2^ Department of Burn and Plastic Surgery, Chongqing University Fuling Hospital, Chongqing, China; ^3^ Central Laboratory of Xinjiang Medical University, Urumqi, China; ^4^ Department of Pathophysiology, School of Basic Medical Sciences, Xinjiang Medical University, Urumqi, China

**Keywords:** WISP1, esophageal squamous cell carcinoma, cancer-associated fibroblasts, tumor microenvironment, immune evasion, extracellular matrix, drug resistance

## Abstract

**Background:**

Previous studies have reported abnormal expression of WNT1-inducible signaling pathway protein 1 (WISP1)/Cellular Communication Network Factor 4 (CCN4) in esophageal squamous cell carcinoma (ESCC). However, its specific significance remains unclear. To date, no in-depth research has been conducted to explore the role and importance of WISP1 in ESCC.

**Methods:**

In this study, we downloaded the expression data of WISP1 (CCN4), Single-Cell RNA Sequencing (scRNA) data, and clinical information from public databases. A combination of bioinformatics analyses and experimental approaches was employed to comprehensively investigate the correlation between WISP1 expression and clinical prognosis, tumor microenvironment (TME), drug resistance, and response to immunotherapy. Additionally, the role of WISP1 in cancer-associated fibroblasts (CAFs) and its underlying mechanisms were explored.

**Results:**

Our findings revealed that WISP1 exhibited differential expression in most analyzed cancers. In ESCC, WISP1 was upregulated and associated with TME characteristics, immune suppression, and drug resistance. Further analysis indicated that ESCC patients with higher WISP1 expression had relatively poorer prognoses. Moreover, it was confirmed that WISP1 is predominantly highly expressed in CAFs. Knockdown of WISP1 in CAFs significantly inhibited their proliferation, migration, and invasion capabilities, as well as markedly reduced the expression of extracellular matrix (ECM) proteins collagen type I alpha 1 chain (COL1A1) and matrix metallopeptidase 14 (MMP14). Notably, co-culture experiments of CAFs with knocked-down WISP1 and ESCC cancer cells demonstrated that the migration and invasion abilities of ESCC cancer cells were also significantly impaired.

**Conclusion:**

In summary, WISP1 is intricately involved in the pathogenesis of ESCC, exhibiting multifaceted roles. WISP1 can modulate the activities of CAFs and cancer cells in ESCC, as well as the process of ECM remodeling, thereby influencing the pathological progression of this malignancy. Based on the aforementioned research findings, WISP1 holds promise as a prognostic molecular marker and a potential therapeutic target for ESCC.

## 1 Introduction

Esophageal cancer (EC) is one of the most prevalent malignancies globally. Notably, there is significant geographical variation in histological subtypes: in high-incidence regions such as China and parts of Asia, ESCC accounts for approximately 90% of EC cases, whereas esophageal adenocarcinoma predominates in Western countries. It ranks 11th in incidence and 7th in mortality among all cancers worldwide (1). Despite recent advancements in clinical treatments for ESCC, patient prognosis remains poor, largely due to the high invasiveness and metastatic potential of EC (2, 3). Therefore, further exploration of molecular mechanisms and identification of novel biomarkers and therapeutic targets are critically important.

WISP1 belongs to the WISP subfamily of CCN matricellular proteins, which plays a key role in ECM remodeling (4, 5). Evidence indicates that WISP1 stimulation can upregulate fibrotic genes such as COL1A1, COL1A2, and Fibronectin1 (FN1) in both mouse and human fibroblasts (6). Additionally, WISP1 can enhance the expression of MMP2, MMP3, and MMP9 in various cell types, including murine macrophages, primary murine renal tubular epithelial cells, primary human chondrocytes, and synovial cells (6, 7). The elevated expression of these ECM proteins promotes ECM remodeling, crosslinking, and deposition, as well as the degradation of specific ECM proteins, thereby fostering a microenvironment conducive to cancer cell proliferation, migration, invasion, and immune evasion (8, 9).

Studies have shown that WISP1 is highly expressed in ESCC tissues and is associated with poor prognosis in ESCC patients (10). WISP1 can act as an autocrine factor to regulate the function of cancer cells (11). Current research demonstrates that WISP1 can activate multiple downstream signaling pathways in various cell types, including epithelial cells, fibroblasts, bone marrow stromal cells, and cancer cells (12). These pathways encompass focal adhesion kinase, RAS/RAF/MEK/ERK, NF-κB, PI3K/AKT, and others. Such mechanisms contribute to tumor cell proliferation, survival, migration/invasion, and metastasis across several malignancies, such as brain, breast, colorectal, lung, pancreatic, and prostate cancers (12).

However, the role and underlying mechanisms of WISP1 in ESCC remain poorly understood. In recent years, bioinformatics has advanced rapidly. The development and refinement of databases, algorithms, and tools have facilitated the integration of bioinformatics approaches into diverse research areas (13, 14), significantly enhancing the identification of disease-driving genes and potential drug targets. In this study, we employed a combination of bioinformatics analyses and experimental methods to investigate WISP1 expression in ESCC, its association with clinical prognosis, its correlation with the TME, drug resistance, and response to immunotherapy, as well as the potential molecular mechanisms involved.

## 2 Materials and methods

### 2.1 Data collection

Microarray gene expression and clinical data for ESCC patients were retrieved from the Gene Expression Omnibus (GEO) database (https://www.ncbi.nlm.nih.gov/geo/) and UCSC Xena (https://xenabrowser.net/datapages/). Specifically, four datasets were utilized in this study: GSE53625, which includes 179 paired cancerous and normal tissue samples; GSE53624, comprising 119 paired samples; GSE161533, consisting of 28 cancerous and 10 normal tissue samples; and TCGA_ESCC, containing 82 cancerous and 11 normal tissue samples. The clinical characteristics of ESCC patients across these datasets are summarized in (**Supplementary Table S1**). Additionally, scRNA-seq data (GSE196756) were obtained from the GEO database, encompassing scRNA-seq profiles from three ESCC and three normal esophageal tissue samples for further analysis.

### 2.2 Identification of DEGs between ESCC and normal tissues

To identify DEGs between ESCC and normal tissues, we employed the “Limma” package (version 3.58.1). This analysis was conducted on the cancerous and normal tissue samples from the four aforementioned microarray gene expression datasets. DEGs were screened using the criteria of adjusted P-value < 0.05 and |log2 fold change (FC)| > 1.

### 2.3 Preliminary investigation of WISP1 gene expression levels

Before performing differential gene expression analysis, we conducted an initial assessment of WISP1 gene expression across multiple cancer types. Using the TIMER2.0 database (http://timer.cistrome.org/), we systematically analyzed WISP1 expression levels in various cancers to elucidate its expression patterns across different tumor types. Additionally, we employed the GEPIA (http://gepia.cancer-pku.cn/) tool to further investigate WISP1 expression specifically in esophageal cancer.

### 2.4 Intersection and functional analysis of DEGs

Following the identification of DEGs from the four datasets, we performed Venn diagram analysis to pinpoint genes that exhibited differential expression across multiple datasets. This approach was undertaken to enhance the reliability and robustness of our findings. Subsequently, a comprehensive functional enrichment analysis was conducted on the intersecting genes using the Gene Ontology (GO, http://geneontology.org/) and Kyoto Encyclopedia of Genes and Genomes (KEGG, https://www.kegg.jp/) databases. This analysis aimed to elucidate the potential roles of these genes in biological processes, cellular components, molecular functions, and signaling pathways.

### 2.5 Co-expression patterns of WISP1 and functional interpretation

To further elucidate the role of WISP1 in ESCC and its interactions with other genes, we conducted an analysis of the co-expression patterns and functional enrichment of WISP1. This was performed using the GeneMANIA platform (https://genemania.org/) and the STRING database (https://cn.string-db.org/).

### 2.6 Prognostic significance of WISP1 expression in ESCC patients

This study was conducted using three independent gene expression datasets (GSE53624, GSE53625, and TCGA), which encompassed the gene expression profiles of ESCC patients along with their corresponding survival data. The optimal cutoff values for stratifying WISP1 expression levels were determined using the x-tile software (version 3.6.1), yielding thresholds of 10.1 for GSE53624, 10.3 for GSE53625, and 1.7 for TCGA. Based on these thresholds, patients were categorized into high WISP1 expression and low WISP1 expression groups. Kaplan-Meier survival curves were subsequently generated to visually compare survival differences between the two groups. To assess the statistical significance of these differences, log-rank tests were performed, and P values were calculated for each Kaplan-Meier (K-M) curve. Furthermore, the prognostic performance of WISP1 expression in ESCC patients was evaluated by constructing receiver operating characteristic (ROC) curves, with the areas under the curve (AUC) calculated to quantify the accuracy of predicting 1-year, 3-year, and 5-year survival outcomes.

### 2.7 Tumor microenvironment and immune cell infiltration analysis

The proportions and spatial distributions of tumor-infiltrating immune cells (TIICs) were quantified using single-sample Gene Set Enrichment Analysis (ssGSEA), implemented via the “GSVA” R package (Version 1.50.5). Additionally, the immune score, stromal score, and ESTIMATE score were calculated utilizing the “ESTIMATE” R package (Version 1.0.13). In addition, we systematically evaluated the expression levels of key inhibitory immune checkpoint molecules (CD274, PDCD1, TIGIT, CD276, CTLA4, LAG3) in groups stratified by high versus low WISP1 expression to identify potential differences. Pairwise Pearson correlation coefficients between WISP1 and immune checkpoint genes were calculated using the cor.test() function in R (version 4.3.2), with statistical significance determined by a two-tailed test. Results were visualized as heatmaps using the ggplot2 package (version 3.4.4).

### 2.8 Functional analysis of DEGs between high-expression and low-expression groups

To elucidate the biological distinctions between the high-expression and low-expression groups of WISP1, we performed a comprehensive functional analysis of DEGs. Specifically, by utilizing the gene sets h.all.v2023.2.Hs.symbols, we systematically investigated the significantly perturbed signaling pathways and functional modules between these two groups through Gene Set Enrichment Analysis (GSEA).

### 2.9 Drug sensitivity analysis

To assess the differences in drug responses between the high-expression and low-expression groups, we employed the R package Oncopredict (Version 1.2) to calculate the sensitivity scores of small-molecule compounds for each patient.

### 2.10 Single-cell RNA sequencing analysis

We initially performed rigorous preprocessing of the raw data using the Seurat software package. By applying a threshold for mitochondrial gene expression percentage (≤20%) and requiring a minimum of 200 expressed genes per cell, we retained 31,293 high-quality cell samples. Subsequently, to account for sequencing depth variability and potential data biases, normalization was conducted using the NormalizeData function. Following this step, we identified 2,000 highly variable genes via the FindVariableFeatures function, which formed a critical feature set for downstream analyses.

To further elucidate the intrinsic structure of the dataset, we applied Uniform Manifold Approximation and Projection (UMAP) for dimensionality reduction. Through UMAP analysis, the cell samples were effectively mapped onto a two-dimensional space, revealing 22 distinct clusters. To accurately annotate the cell types within these clusters, we performed a comprehensive analysis leveraging known biomarkers. Using specific markers such as EPCAM and keratin genes, we successfully identified various cell populations, including epithelial/tumor cells, T cells, myeloid cells, B cells, endothelial cells, fibroblasts, and neutrophils (15).

Following the identification of cell types, we further employed the FindAllMarkers function to calculate DEGs. By applying stringent filtering criteria, we successfully identified genes with significant expression differences, which provided critical insights for subsequent functional analyses. Specifically, for fibroblasts—a key cell type—we stratified them into two subpopulations based on WISP1 expression levels: high-expression and low-expression groups. We subsequently calculated the DEGs between these subpopulations. To elucidate the biological significance of these DEGs, we applied GSEA to assess their associations with canonical biological functions and regulatory mechanisms.

Finally, to explore the interactions and communication networks among various cell types, we conducted intercellular communication analysis using the “CellChat” R package (Version 1.6.1). This analysis not only enhanced our understanding of the complexity and dynamics within the TME but also furnished valuable references for the development of future therapeutic strategies.

### 2.11 Esophageal tissue samples from ESCC patients

In this study, all human ESCC tissue samples were obtained from patients at the First Affiliated Hospital of Xinjiang Medical University. We collected 12 ESCC tissue samples from patients who underwent surgical resection, some of which were processed with paraffin embedding. All participating patients provided informed consent, and the study was officially approved by the Ethics Review Committee of the First Affiliated Hospital of Xinjiang Medical University.

### 2.12 Isolation and culture of fibroblasts

Tumor tissues and adjacent non-tumor tissues were collected from ESCC patients, and CAFs as well as corresponding normal fibroblasts (NFs) were isolated from these specimens. The procedure was conducted as follows: Initially, the tissues were minced into 1–2 mm pieces in sterile PBS. They were subsequently digested with 0.25% trypsin for 30 minutes at room temperature, with intermittent shaking, followed by centrifugation at 1000 rpm for 5 minutes to remove residual trypsin. Next, digestion was continued using a mixed collagenase solution (CTCC, 008PI) for 2 hours under the same shaking conditions. Afterward, the samples were centrifuged again at 1000 rpm for 5 minutes, and the supernatant was discarded. The resulting cell pellets were resuspended in complete human fibroblast medium (CM-H103, Pricella, China) and plated in culture dishes, which were incubated at 37°C in a humidified atmosphere containing 5% CO_2_. To enrich for pure fibroblast populations, the cells were subjected to pre-culture at 30°C for 30 minutes to eliminate non-adherent cells, primarily tumor cells.

### 2.13 ESCC cell culture

The ESCC cell lines KYSE-150 and Eca109 were obtained from Procell Life Technology Co., Ltd. (Wuhan, China) and authenticated through STR profiling and mycoplasma testing. These cells were maintained in RPMI-1640 medium supplemented with 10% fetal bovine serum (FBS), 100 U/mL penicillin, and 100 μg/mL streptomycin. Cultures were incubated at 37°C under a humidified atmosphere containing 5% CO_2_.

### 2.14 Immunohistochemistry

The immunohistochemical analysis was performed as follows: Paraffin-embedded tissue sections were deparaffinized with xylene, rehydrated through a graded ethanol series, and treated with 3% hydrogen peroxide for 10 minutes to inhibit endogenous peroxidase activity. Antigen retrieval was achieved by heating the sections in EDTA buffer, followed by blocking with normal goat serum to reduce non-specific binding. The sections were subsequently incubated overnight at 4°C with the primary antibody against WISP1 (diluted at 1:100). After washing, the sections were incubated with the corresponding secondary antibody for 30 minutes at 37°C. Color development was carried out using 3,3’-diaminobenzidine (DAB), followed by counterstaining with hematoxylin, dehydration, and mounting. The staining results were evaluated by a certified pathologist, who assessed both the number of positive cells and the staining intensity. The immunoreactivity score (IRS) was then calculated based on these parameters.

### 2.15 Lentiviral transfection of CAFs

The lentiviral vector was constructed by Genechem Co., Ltd. (Shanghai, China) for transfection into CAFs cells. The vector contained the shRNA sequence targeting human CCN4/WISP1 (5’-GCATCCATGAACTTCACACTT-3’) and included a negative control shRNA. A multiplicity of infection (MOI) of 50 was used during transfection. The knockdown efficiency of WISP1 was verified by qRT-PCR and Western blot analysis.

### 2.16 Quantitative real-time PCR analysis

Total RNA was isolated from cells using TRIzol reagent (Takara Bio Inc., Japan). Reverse transcription was subsequently performed with the ReverTra Ace™ qPCR RT Kit and Master Mix (Toyobo, Japan). Quantitative real-time PCR was conducted using SYBR Green technology on the QuantStudio™ 3 Real-Time PCR System (Applied Biosystems, USA). mRNA expression levels were normalized to GAPDH as an internal control and are presented as relative fold changes compared to those in the control group. The primer sequences for the target genes are summarized in (**Supplementary Table S2**).

### 2.17 Western blot and phospho-kinase array

Primary fibroblasts were infected with shWISP1 lentivirus or treated with recombinant human WISP1 (rhWISP1) protein(NP_003873.1, Sino Biological, China; endotoxin level <0.1 EU/μg, as determined by Limulus Amebocyte Lysate assay), followed by three washes with PBS. Total protein extraction was performed using a lysis buffer supplemented with protease and phosphatase inhibitors. Protein concentrations were quantified using a BCA assay kit. Proteins were subsequently separated by 10% SDS-PAGE and transferred onto PVDF membranes. The membranes were blocked with 5% non-fat milk at room temperature for 2 hours and then incubated with primary antibodies overnight at 4°C. On the following day, the membranes were incubated with secondary antibodies at room temperature for 1 hour, followed by chemiluminescent detection using an ECL reagent. Detailed information on the antibodies is provided in (**Supplementary Table S3**). Phosphorylation profiling was conducted with the Proteome Profiler Human Phospho-Kinase Array Kit (R&D Systems,ARY003C). Cellular proteins were extracted using RIPA lysis buffer, followed by centrifugation at 16,900 × g for 10 minutes (4°C). Subsequent procedures strictly adhered to the manufacturer’s specifications.

### 2.18 Enzyme-linked immunosorbent Assay

Cells (CAFs, CAFs-shVector, CAFs-shWISP1) were seeded into 96-well plates at a density of 5 × 10³ cells per well in 100 μL of RPMI 1640 medium supplemented with 10% fetal bovine serum (FBS). After 12 hours of incubation for cell attachment, the initial medium was replaced with 200 μL of fresh complete medium. Each experimental group was set up in triplicate wells. Following a 1-hour equilibration period, culture medium from the three wells of each group was collected as the 0-hour sample. The remaining wells were further incubated for 24 and 48 hours, respectively, after which the culture supernatants were harvested. All collected samples were centrifuged at 3000 × g for 20 minutes prior to analysis. WISP1 levels in the culture supernatants were then quantified using an Elisa (ml038498, Shanghai Enzyme-linked Biotechnology Co., Ltd., China), according to the manufacturer’s instructions. The detection range of the assay is 12.5 pg/mL to 400 pg/mL.

### 2.19 Edu cell proliferation assay

CAF-Vector and CAF-shWISP1 cells were seeded at equal densities into 6-well plates and cultured for 48 hours. Thereafter, cell proliferation was assessed using the BeyoClick™ EdU-594 Cell Proliferation Detection Kit (Product No. C0078S, Beyotime Biotechnology, China). Briefly, cells were fixed with 4% paraformaldehyde and subjected to EdU labeling according to the manufacturer’s protocol.

### 2.20 Cell counting kit-8

CAFs-shVector cells and CAFs-shWISP1 cells in the logarithmic growth phase were seeded into 96-well plates at a density of 2,000 cells per well. For each sample, six replicate wells were established, and blank control wells (containing only culture medium) were also set up. Subsequently, the 96-well plates were incubated in an incubator at 37°C with 5% CO_2_. At the time points of 0 hours, 12 hours, 24 hours, 48 hours, and 72 hours post-seeding, 100μL of CCK-8 reagent (10% concentration) was added to each well, followed by further incubation of the plates in the incubator for 90 minutes. After incubation, the absorbance (optical density, OD) of each well at 450 nm was measured using a Microplate Reader (model Varioskan LUX, manufactured by Thermo Fisher Scientific, USA).

Logarithmic phase CAFs was seeded as described above. Stattic was dissolved in DMSO to prepare stock solutions at various concentrations, which were then diluted in the culture medium to create a series of working solutions (1-15 μM). Different concentrations of Stattic working solutions or DMSO were added to the 96-well plates containing cells, ensuring a final volume of 100 μL per well. The plates were incubated for 48 hours in the incubator. Subsequent CCK8 assays were performed as previously described. Finally, the relative cell viability was calculated based on the OD values of CAFs treated with different concentrations of Stattic (relative cell viability = (OD value of treatment group - OD value of blank control)/(OD value of negative control - OD value of blank control) × 100%). Dose-response curves were generated using GraphPad Prism (version 6.01), and the IC50 values of Stattic for CAFs was determined.

### 2.21 Immunofluorescence staining procedure

CAFs cultured on coverslips were fixed with 4% paraformaldehyde for 15 minutes at room temperature upon reaching 70%–80% confluency. Following fixation, the samples were washed three times with PBS buffer for 5 minutes each. Cell permeabilization was then performed using 0.5% Triton X-100 (P0096, Beyotime, China) for 10 minutes at room temperature. To minimize non-specific antibody binding, antigen blocking was carried out by incubating the samples in a PBS solution containing 3% BSA for 30 minutes. Subsequently, the samples were incubated with primary antibodies at 37°C for 2 hours. Further steps were performed according to the manufacturer’s instructions provided in the Dual-color Multiplex Fluorescence Staining (TSA) Kit (AFIHC023, Aifang Biological, China). Finally, fluorescence images were acquired using a Leica DFC7000T microscope. Detailed information regarding the sources and dilutions of the antibodies used is provided in (**Supplementary Table S3**).

### 2.22 Migration and invasion assays

For migration assays, Transwell chambers (8μm pore size; Corning, USA) without Matrigel coating were employed. For invasion assays, Matrigel (356234, Corning, USA) was evenly applied to the surface of the Transwell inserts and incubated at 37°C for 2 hours to allow gel polymerization. CAFs or cancer cells were seeded in the upper chamber at a density of 1 × 10_5_ cells/ml in serum-free RPMI-1640 medium, while the lower chamber contained RPMI-1640 medium supplemented with 10% fetal bovine serum (FBS) as a chemoattractant. Alternatively, CAFs were directly seeded at the same density. The cells were then incubated at 37°C in a humidified atmosphere with 5% CO_2_ for 48 hours. Following incubation, the cells were fixed with 4% paraformaldehyde, and non-migrated or non-invaded cells on the upper surface of the membrane were gently removed using a cotton swab. Cells that had migrated or invaded to the lower surface of the membrane were stained with 0.1% crystal violet, washed with distilled water, and visualized under a light microscope. Images were captured at three random fields of view (magnification ×20), and the number of migrated or invaded cells was quantified.

### 2.23 Statistical analysis

All data analyses were performed using R software (version 4.3.2) and GraphPad Prism (version 6.01). Univariate Cox regression analysis, coupled with the Log-rank test, was employed to assess differences in survival outcomes among groups. The Wilcoxon rank-sum test was utilized for non-parametric comparisons of immune scores, stromal scores, Estimate scores, and drug sensitivity scores between distinct groups. In addition, Student’s t-test was applied where appropriate for specific data analyses. For all statistical tests, a two-sided P-value threshold of less than 0.05 was adopted to determine statistical significance.

## 3 Results

### 3.1 Expression of WISP1 in ESCC from a pan-cancer perspective and its correlation with patient clinical prognosis

**Figure 1:**
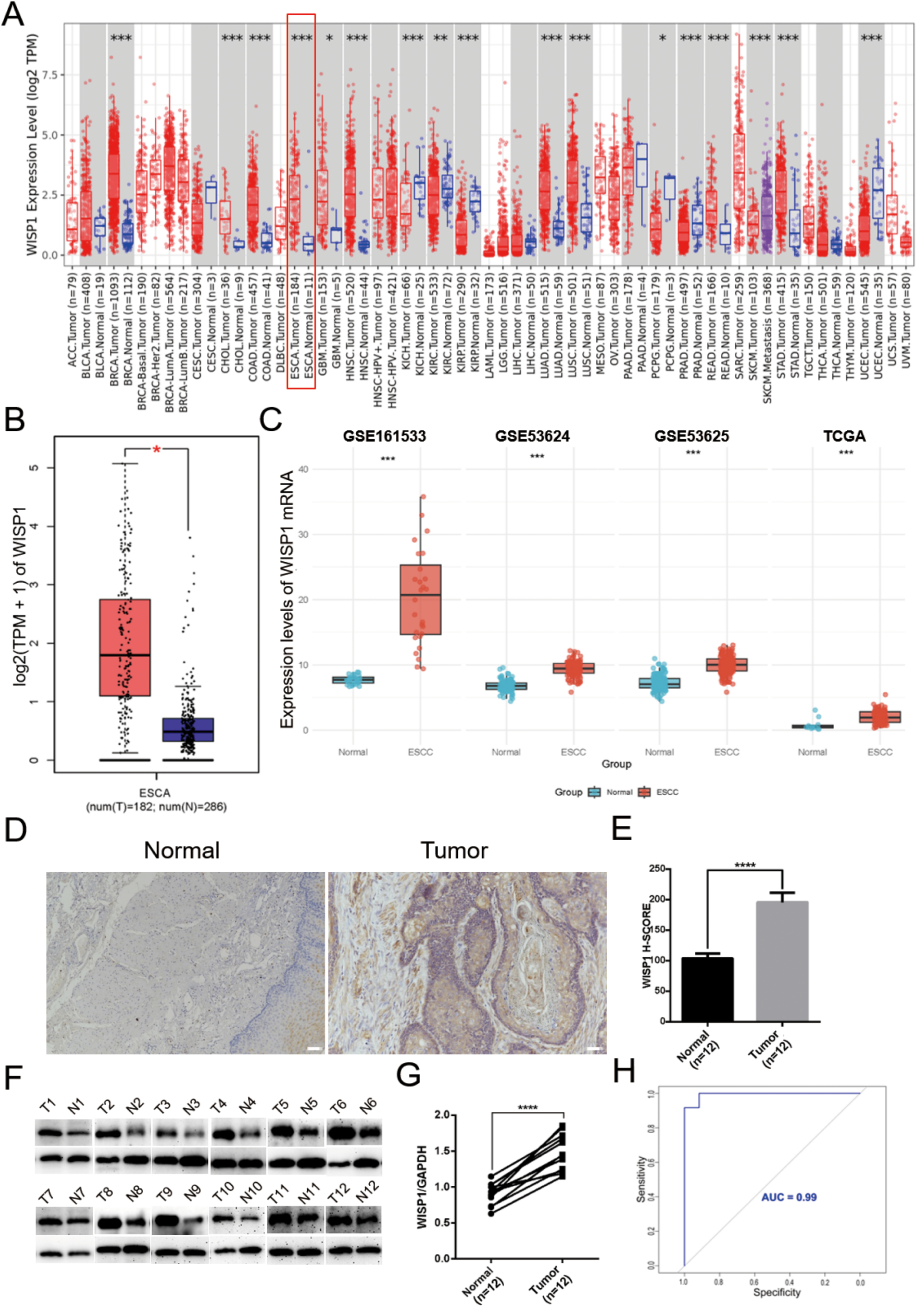
Differential expression of WISP1 in normal and tumor tissue samples. **(A)** Expression of WISP1 in pan-cancer tissues and adjacent normal tissues via TIMER2.0. **(B)** Expression levels of WISP1 in ESCA from GEPIA2 (|Log2FC|> 1, P < 0.01, log scale: log2 (TPM + 1), Jitter Size: 0.4; T: Tumor, N: Normal). **(C)** Expression of WISP1 mRNA in the ESCC dataset. **(D–G)** Results of IHC and WB analyses using 12 paired ESCC tissues and adjacent control samples (T: Tumor, N: Normal; scale bars = 100μm). **(H)** ROC curves predicting the prognostic ability of high WISP1 expression for 1-year, 3-year, and 5-year patient survival. *p < 0.05; **p < 0.01; ***p < 0.001,;****P < 0.0001.

To gain a comprehensive understanding of the expression patterns of WISP1 across various cancer types, we initially evaluated its expression levels in 33 distinct cancer types using the TIMER2.0 database. Our analysis revealed that WISP1 exhibited significant differential expression in most cancer types (**Figure 1A**). To further validate these findings, we integrated data from the GEPIA database, which confirmed that WISP1 expression was markedly upregulated in EC (P<0.05) (**Figure 1B**). Considering that our research focuses on ESCC, we conducted an in-depth analysis of four datasets containing ESCC-related data (GSE53624, GSE53625, GSE161533, and TCGA). Although the WISP1 expression levels in the ESCC group within the GSE161533 dataset were significantly higher than those in the other datasets, this discrepancy may be attributed to its smaller sample size or differences in technical platforms. Nevertheless, consistent results across all datasets demonstrated that WISP1 expression in ESCC tissues was significantly higher than in normal esophageal tissues, confirming its key role in ESCC (P<0.001) (**Figure 1C**).

To further validate these findings, we conducted IHC and WB experiments. The results demonstrated a significant upregulation of WISP1 expression in ESCC tissues compared to normal esophageal tissues (P<0.001)(**Figures 1D–G**). Moreover, we assessed the diagnostic potential of WISP1 by performing ROC curve analysis. This analysis resulted in an AUC value of 0.99, underscoring the high accuracy of WISP1 as a biomarker for differentiating between tumor and normal tissues (**Figure 1H**).

**Figure 2:**
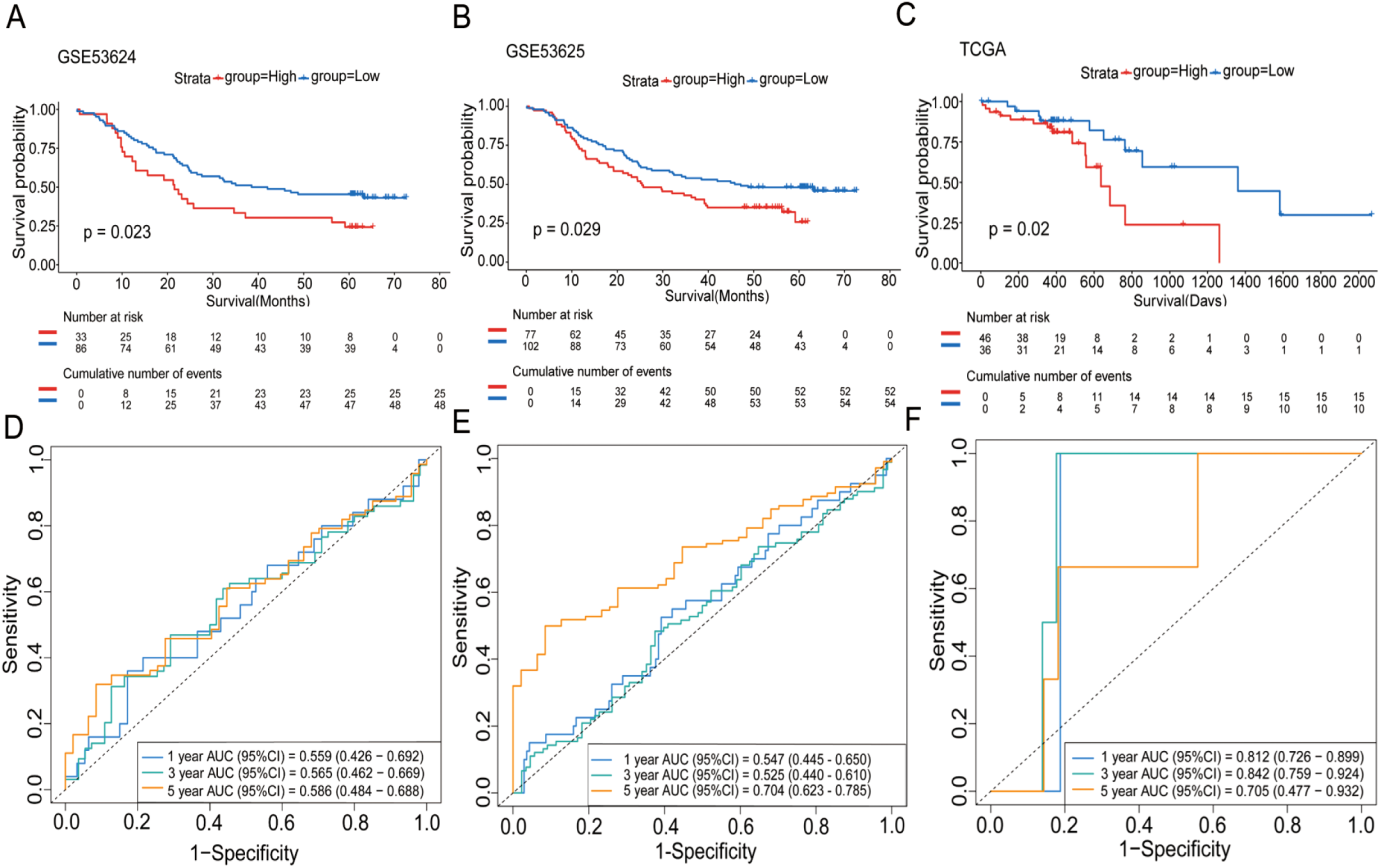
The impact of WISP1 expression on the survival of ESCC patients. **(A–C)** Kaplan-Meier analysis of overall survival for high-expression versus low-expression groups in the datasets GSE53624, GSE53625, and TCGA. **(D–F)** Time-dependent ROC analysis for patients in the datasets.

In our subsequent analyses, we aimed to investigate the correlation between WISP1 expression and patient prognosis. Using K-M survival curve analyses, we evaluated three datasets encompassing patient prognostic information (GSE53624, GSE53625, and TCGA) (**Supplementary Tables S4**–**S6**, P<0.05). The findings indicated that patients in the high WISP1 expression group exhibited markedly reduced survival times, thereby establishing a robust association between elevated WISP1 expression and adverse clinical outcomes in ESCC patients (**Figures 2A–C**).

Finally, we assessed the prognostic performance of WISP1 expression for 1-year, 3-year, and 5-year survival in three datasets using ROC curve analysis. Specifically, the AUC values for the GSE53624 dataset were 0.559, 0.565, and 0.586, respectively; for the GSE53625 dataset, they were 0.547, 0.525, and 0.704; and for the TCGA dataset, the AUC values were 0.812, 0.842, and 0.705 (**Figures 2D–F**). While the AUC values for 1-year and 3-year predictions were relatively modest in some datasets, the overall results suggest that WISP1 expression holds potential as a predictor of survival outcomes in ESCC patients.

### 3.2 Identification and functional enrichment analysis of DEGs in ESCC and functional annotation of WISP1

**Figure 3:**
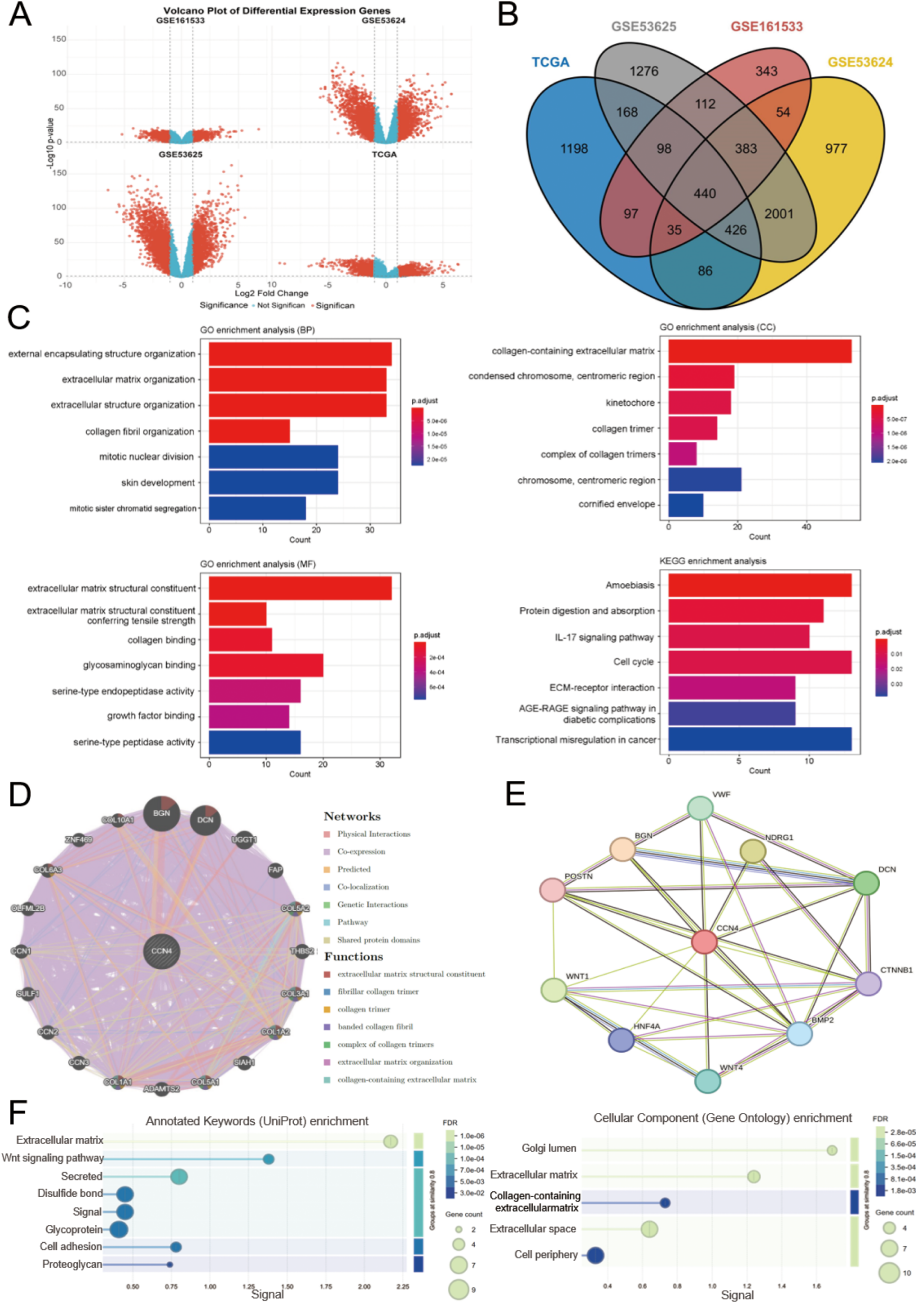
Identification of DEGs in ESCC, functional enrichment analysis, and functional annotation of WISP1. **(A)** Volcano plot shows the DEGs from four datasets. **(B)** A Venn diagram shows genes that are differentially expressed across all four datasets. **(C)** GO and KEGG analyses reveal the potential biological mechanisms of DEGs in the four datasets. **(D, E)** GeneMANIA and STRING databases identify target proteins and genes associated with WISP1, followed by enrichment analysis of the functions of these proteins and genes. **(F)** Visualization of enrichment analysis results.

We initially screened for DEGs in four transcriptomic datasets related to ESCC, namely GSE161533, GSE53624, GSE53625, and TCGA. The screening criteria were set at |log2(fold change)| ≥ 1 and p < 0.05 (**Supplementary Tables S7**-**S10**, **Figure 3A**). Using Venn diagram analysis, we identified a total of 440 overlapping DEGs across these datasets (**Supplementary Table S11**, **Figure 3B**). GO and KEGG functional enrichment analyses were performed to explore the underlying biological mechanisms associated with these DEGs.

The GO analysis results revealed that these DEGs are predominantly involved in the organization and structural maintenance of the ECM. Additionally, KEGG pathway analysis highlighted several key pathways, including “Transcriptional misregulation in cancer,” “Amoebiasis,” “Cell cycle,” and “ECM-receptor interaction” (**Figure 3C**). These findings suggest that alterations in ECM-related processes and cellular signaling pathways may play critical roles in ESCC progression.

To gain deeper insights into the functional network and biological roles of WISP1, we conducted protein-protein interaction (PPI) analyses using the GeneMANIA and STRING databases. These platforms identified potential interacting partners of WISP1 and reconstructed the associated interaction networks. Subsequently, we performed functional enrichment analysis on these interacting proteins to elucidate their involvement in key biological processes and molecular functions. This integrative approach offers a systematic understanding of the mechanistic roles of WISP1 in disease progression. (**Figures 3D, E**). Our results demonstrate that WISP1 is significantly involved in the organization and structural integrity of the ECM (**Figure 3F**). This finding is highly consistent with the known biological features of ESCC and offers new insights into the mechanisms by which WISP1 contributes to the development and progression of ESCC. These findings highlight the potential of WISP1 as a candidate biomarker or therapeutic target in ESCC research.

### 3.3 The impact of WISP1 expression on the tumor microenvironment

**Figure 4:**
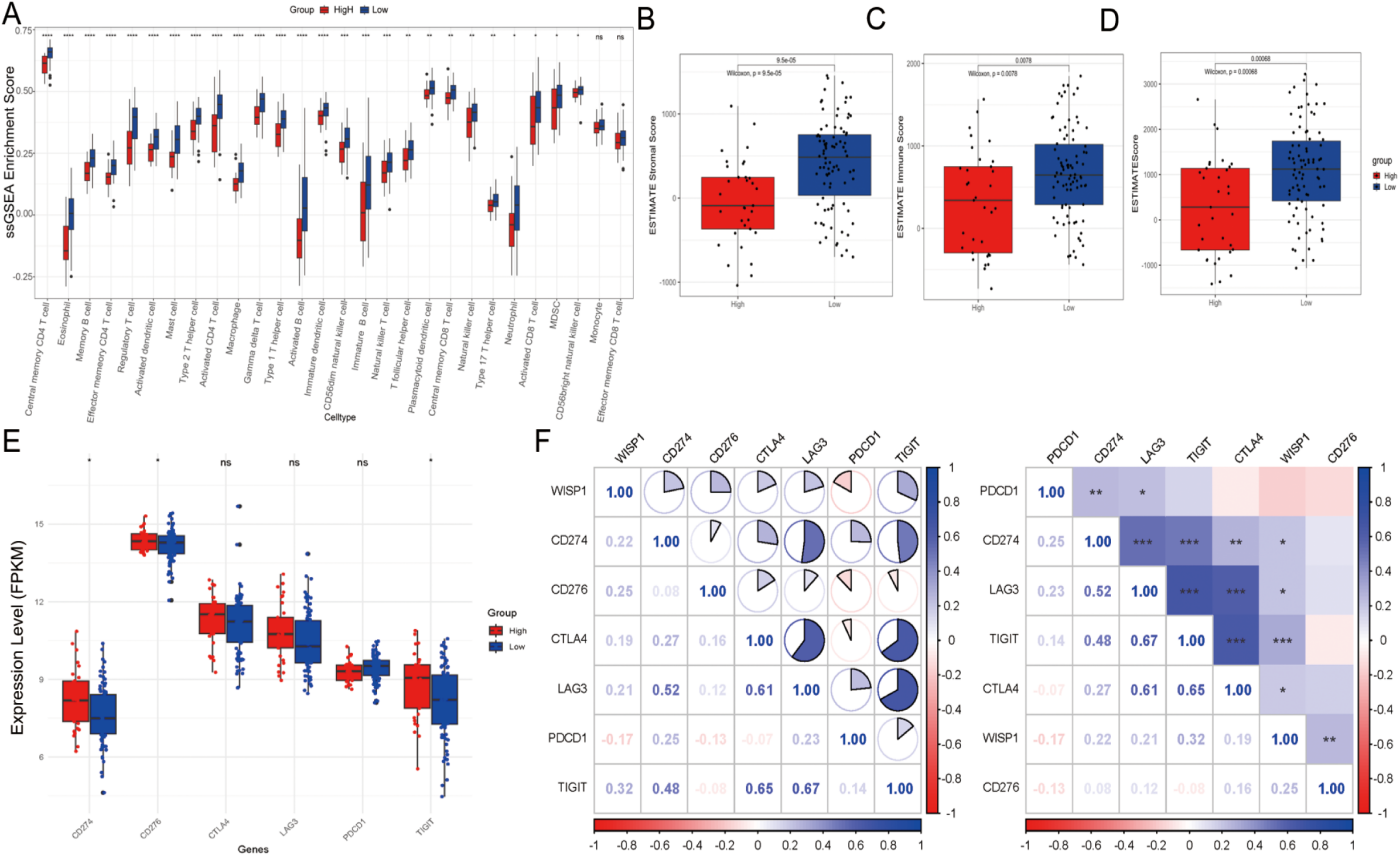
Tumor microenvironment and immune cell infiltration analysis in the GSE53624 dataset. **(A)** Differences in the abundance of infiltrating immune cells between high-expression and low-expression groups. **(B–D)** Differences in stromal scores, immune scores, and estimate scores between high-expression and low-expression groups. **(E)** Evaluation of the expression of immune checkpoint molecules (CD274, PDCD1, TIGIT, CD276, CTLA4, LAG3) between high-risk and low-risk groups. **(F)** Correlation between WISP1 expression and immune checkpoint molecules (CD274, PDCD1, TIGIT, CD276, CTLA4, LAG3). Blue indicates positive correlation, red indicates negative correlation, and the numbers inside the boxes represent the magnitude of the correlation. *p < 0.05; **p < 0.01; ***p < 0.001; ****P < 0.0001.

To further elucidate the potential mechanisms underlying the clinical prognostic disparities between the high-expression and low-expression groups, we assessed the immune score, stromal score, and ESTIMATE score. Our findings revealed that in the high-expression group, the infiltration levels of most immune cell subsets were significantly downregulated, with the exception of monocytes and effector memory CD8-positive T cells, which showed no significant differences in infiltration (**Figure 4A**). This observation indicates the presence of an immunosuppressive microenvironment. Moreover, both the immune score, stromal score, and ESTIMATE score were markedly reduced in the high-expression group, suggesting a less favorable tumor microenvironment (P < 0.001) (**Figures 4B–D**). Further analysis demonstrated that immune checkpoint inhibitory molecules, including CD274/PDL1 (P < 0.05), CD276 (P < 0.05), and TIGIT (P < 0.05), were upregulated in the high-expression group, potentially exacerbating immune escape mechanisms (**Figure 4E**). Notably, WISP1 expression exhibited a positive correlation with these molecules, implicating its potential regulatory role (**Figure 4F**). In an additional dataset (GSE53625), the high-expression group also displayed similar trends, with a decreasing trend in immune scores, although this did not reach statistical significance (P = 0.1). However, both the stromal and ESTIMATE scores were significantly reduced (P < 0.001) (**Supplementary Figures S1A–D**). Furthermore, CD274/PDL1 expression was significantly upregulated (P < 0.001), whereas PDCD1 (P < 0.001), CD276 (P < 0.001), CTLA4 (P < 0.01), and LAG3 (P < 0.01) expressions were significantly downregulated (**Supplementary Figure S1E**). WISP1 exhibited a positive correlation with CD274/PDL1 but a negative correlation with the other genes (**Supplementary Figure S1F**).

In summary, the poorer clinical outcomes observed in the high-expression group may be associated with an impaired immune microenvironment, characterized by reduced immune cell infiltration, lower scores, and upregulation of immune checkpoint inhibitory molecules. These findings suggest that WISP1 may play a critical role in modulating the TME.

### 3.4 The high expression of WISP1 in ESCC is significantly associated with the activation of the epithelial-mesenchymal transition process

**Figure 5:**
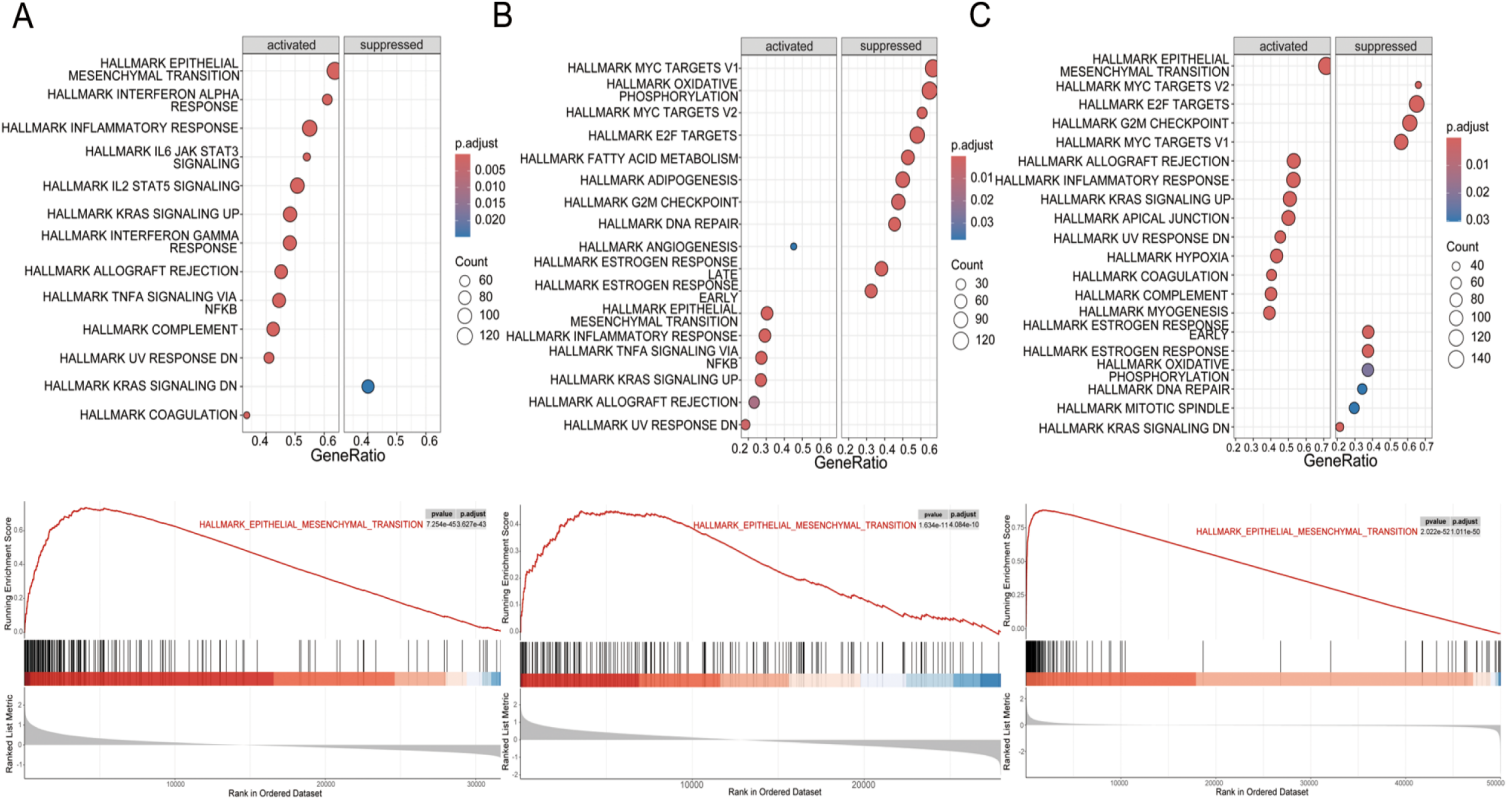
Gene set enrichment analysis (GSEA) of hallmark pathways for esophageal squamous cell carcinoma (ESCC) patients stratified by WISP1 expression levels. **(A–C)** Enrichment patterns of associated pathways in the GSE53624, GSE53625, and TCGA datasets. The analyses were evaluated using a significance threshold corrected for the False Discovery Rate (FDR).

To further investigate this association, we conducted GSEA on high and low WISP1 expression groups using the GSE53624, GSE53625, and TCGA datasets. The results demonstrated that the Hallmark EMT pathway was consistently the most significantly activated pathway in the high WISP1 expression group across all three datasets (**Figures 5A–C**). Notably, in the GSE53625 and TCGA datasets, several proliferation-related pathways, including Hallmark E2F Targets, Hallmark MYC Targets V1, and Hallmark MYC Targets V2, were found to be suppressed in the high WISP1 expression group (**Figures 5B, C**). Collectively, these findings indicate that elevated WISP1 expression plays a pivotal role in promoting the EMT process in ESCC patients.

### 3.5 The association between high WISP1 expression and drug resistance in ESCC

**Figure 6:**
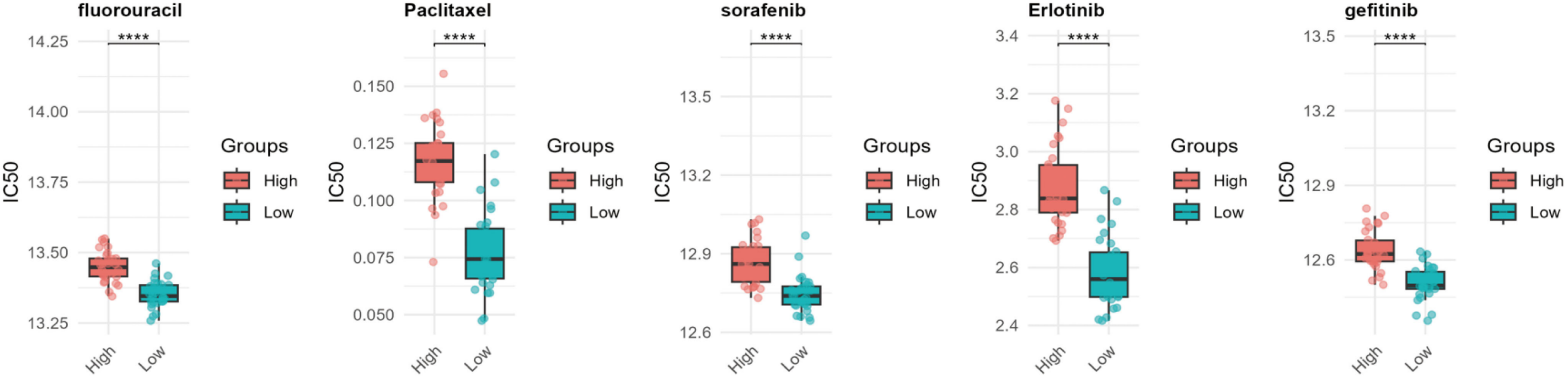
Computational prediction of drug sensitivity for ESCC patients stratified by WISP1 expression levels. Using WISP1 expression grouping derived from the GSE53624 dataset, the “oncoPredict” R package was employed to evaluate the drug response spectrum. **** indicates statistical significance at p < 0.0001.

Drug resistance remains a major obstacle in cancer therapy, significantly hindering treatment efficacy and patient outcomes (16). To explore the potential role of WISP1 in drug resistance, we utilized the “oncoPredict” package in R software, combined with WISP1 expression grouping data derived from the GSE53624 dataset, to evaluate the correlation between WISP1 expression levels and drug responses in ESCC. Our findings reveal a significant association between elevated WISP1 expression and reduced tumor sensitivity to multiple therapeutic agents (**Supplementary Table S12**, **Figure 6**). These results highlight WISP1 as a potential biomarker for predicting drug resistance in ESCC.

### 3.6 Specific expression patterns of the WISP1 gene in different cell types within the ESCC microenvironment

To explore the expression profiles of the WISP1 gene and its role in cellular heterogeneity within the ESCC microenvironment, we performed single-cell RNA sequencing (scRNA-Seq) analysis on the GSE196756 dataset. Following stringent quality control steps—including removal of low-quality cells (nFeature_RNA < 200 or >7500; mitochondrial gene percentage > 20%), data normalization using LogNormalize (scale factor = 10,000), and selection of the top 2,000 highly variable genes—31,293 high-quality single-cell samples were retained for further analysis. Cell types were annotated using enrichment scoring methods based on established marker genes for various cell populations (15).

The results revealed that the samples predominantly comprised epithelial cells (including normal esophageal epithelial cells and tumor cells), immune cells (such as myeloid_cells, mast_cells, T_cells, B_cells, and neutrophils), fibroblasts, and endothelial_cells (**Supplementary Figures S2A–C**). Differential expression analysis was conducted using the FindAllMarkers function to identify marker genes for each cell subpopulation (**Supplementary Figure S2D**). Notably, WISP1 was found to be significantly upregulated in fibroblasts within the ESCC microenvironment (**Supplementary Figure S2E**). Additionally, canonical CAF markers ACTA2 and FAP exhibited elevated expression levels in tumor samples (**Supplementary Figure S2E**). These findings suggest that WISP1 may play a critical role in CAF biology and confirm the accuracy of cell type classification. Together, these results provide a solid foundation for further investigations into the functional mechanisms of WISP1 in ESCC progression.

### 3.7 Function and expression pattern of WISP1 in fibroblasts

**Figure 7:**
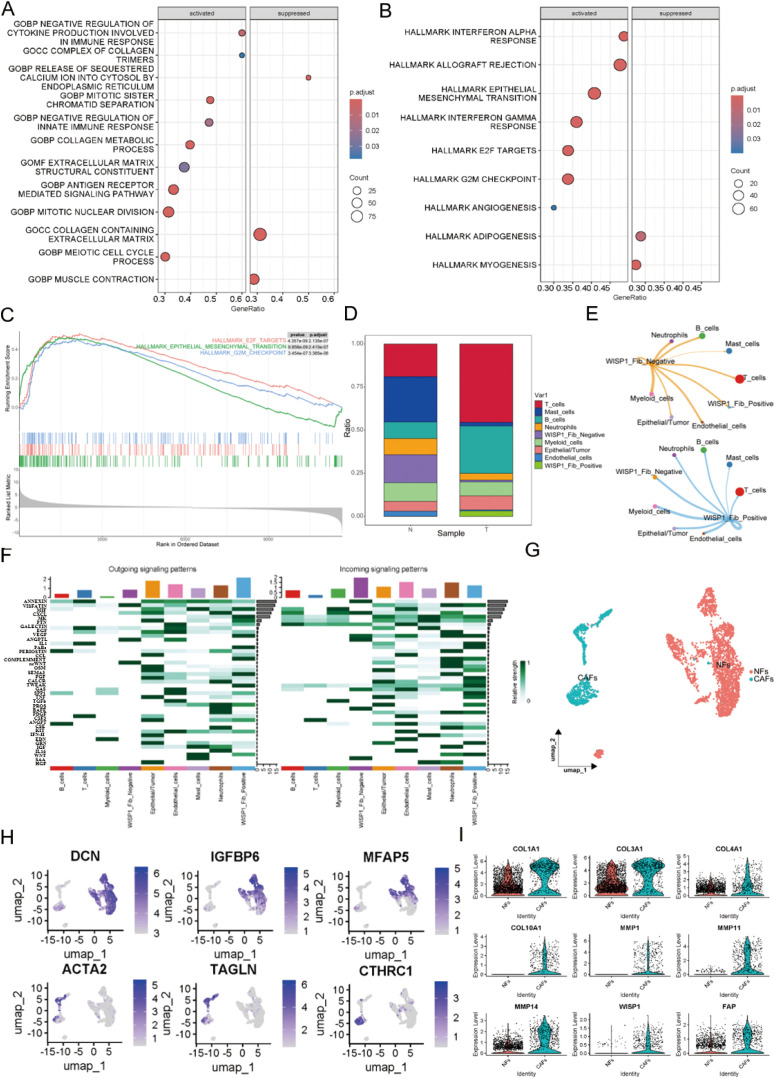
Functional characterization of WISP1 in a subpopulation of fibroblasts. **(A)** Gene Set Enrichment Analysis (GSEA) shows enrichment results of differentially expressed genes between WISP1_Fib_Negative and WISP1_Fib_Positive clusters within the c5.all.v2024.1.Hs.symbols gene set; **(B, C)** display the enrichment results of DEGs in the h.all.v7.1.symbols gene set. **(D)** Proportional distribution of WISP1_Fib_Negative and WISP1_Fib_Positive clusters in normal esophageal tissues and ESCC samples. **(E, F)** Comparative analysis of intercellular communication signal intensity among fibroblast subpopulations. **(G, H)** Molecular stratification of fibroblasts using lineage-specific markers (DCN/Decorin, IGFBP6/Insulin Like Growth Factor Binding Protein 6, MFAP5/Microfibril Associated Protein 5, ACTA2/Actin Alpha 2 Smooth Muscle, TAGLN/Transgelin, CTHRC1/Collagen Triple Helix Repeat Containing 1)into NFs and CAFs subtypes. **(I)** Validation of CAF-specific markers (FAP, COL1A1, COL3A1, COL4A1, COL10A1, MMP1, MMP11, MMP14) and expression patterns of WISP1 in CAFs via single-cell RNA sequencing.

To explore the potential impact of WISP1 on fibroblast function, we classified fibroblasts into two distinct clusters based on WISP1 expression levels: WISP1_Fib_Negative and WISP1_Fib_Positive. After identifying DEGs between these clusters, GSEA was performed (**Supplementary Figure S2F**). Analysis using the c5.all.v2024.1.Hs.symbols gene set revealed that the predominant activated gene sets were associated with ECM structure and function, as well as immune regulation (**Figure 7A**). In contrast, the h.all.v7.1.symbols gene set highlighted activation of gene sets linked to EMT, E2F targets, and G2M checkpoint-related pathways (**Figures 7B, C**).

Furthermore, cell proportion statistical analysis showed that the WISP1_Fib_Negative cluster predominated in normal esophageal tissue samples. In contrast, the WISP1_Fib_Positive cluster tended to aggregate more in ESCC tissue samples, with its proportion significantly lower than that of the WISP1_Fib_Negative cluster (**Figure 7D**). Additionally, cell communication analysis demonstrated that the signal intensity emitted by the WISP1_Fib_Positive cluster was significantly higher than that of the WISP1_Fib_Negative cluster (**Figures 7E, F**).

To more accurately delineate fibroblast heterogeneity, we employed specific markers for NFs and CAFs to further stratify the fibroblast population (17) (**Figures 7G, H**).The specific marker FAP and various ECM proteins (COL1A1, COL3A1, COL4A1, COL10A1, MMP1, MMP11, MMP14) were found to be highly expressed in CAFs, thereby validating the accuracy of our fibroblast classification. More importantly, we also detected robust expression of WISP1 in CAFs (**Figure 7I**). This observation not only expands our understanding of the expression profile of WISP1 but also highlights its potential role in the functional regulation of CAFs.

### 3.8 Identification of NFs and CAFs in ESCC samples and differential WISP1 expression in these populations

**Figure 8:**
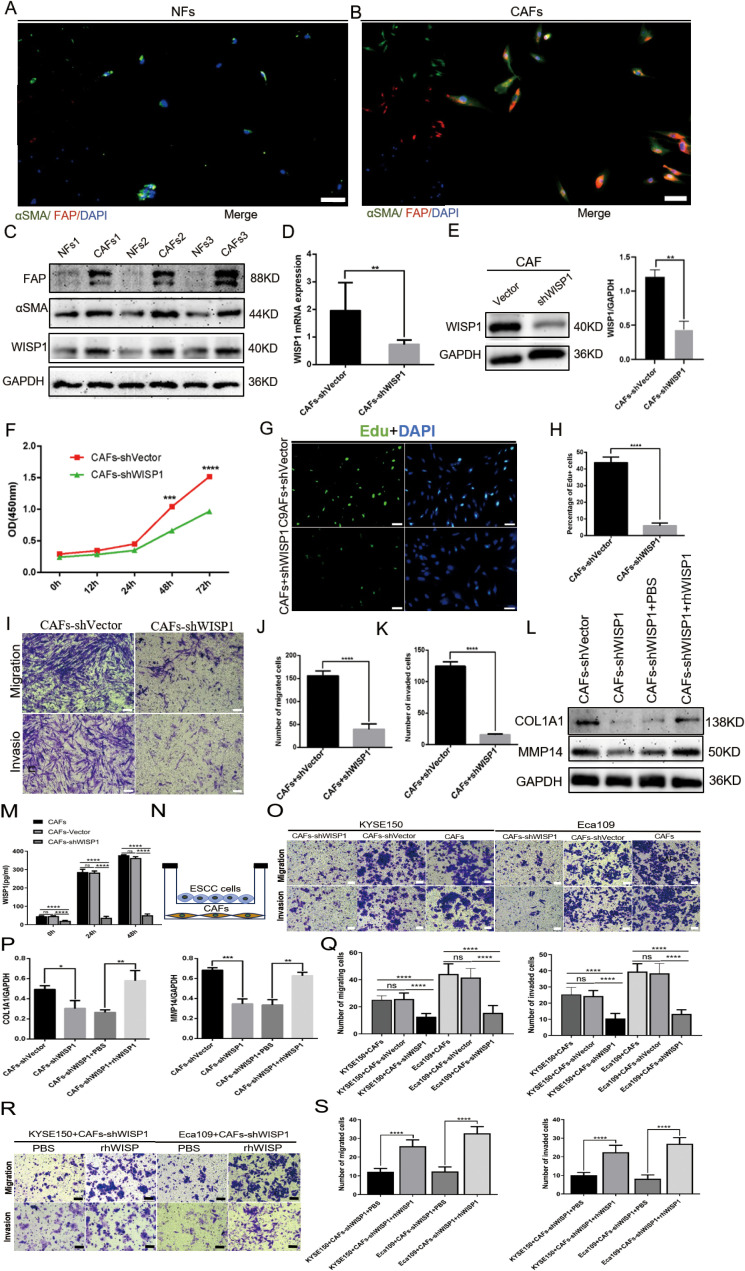
WISP1 regulates cancer-associated fibroblast function and extracellular matrix remodeling. **(A, B)** Immunofluorescence multiplex staining of α-smooth muscle actin (αSMA) and fibroblast activation protein (FAP) in paired cancer-associated fibroblasts (CAFs, tumor-derived) and normal fibroblasts (NFs, adjacent non-tumor tissues) (scale bars = 50μm). **(C)** Western blot (WB) analysis of WISP1, αSMA, and FAP expression in CAFs versus NFs. **(D, E)** Lentiviral short hairpin RNA (shRNA)-mediated WISP1 knockdown in CAFs, validated by quantitative reverse transcription PCR (qRT-PCR) and WB. **(F–K)** Functional characterization of CAF proliferation (CCK-8/EdU), migration, and invasion (Transwell) post-WISP1 silencing (scale bars = 50μm). **(M)** ELISA quantification of secreted WISP1 in supernatants from untransfected CAFs, CAFs-shVector, and CAFs-shWISP1 at 0 h, 24 h, and 48 h **(N)** Schematic of indirect co-culture system for CAF-ESCC interaction analysis. **(L, P)** WB assessment of extracellular matrix (ECM)-remodeling markers (COL1A1, MMP14) in WISP1-depleted CAFs and rescue via recombinant human WISP1 (rhWISP1). **(O, Q)** Transwell assay comparing the migration and invasion capacities of KYSE150 (left) and Eca109 (right) cells co-cultured with untransfected CAFs, CAFs-shVector, or CAFs-shWISP1(scale bars = 50μm). **(R, S)** Transwell assay assessing the migration and invasion capacities of KYSE150 (left) and Eca109 (right) cells in the CAFs-shWISP1 co-culture system following rescue experiments with rhWISP1 supplementation (scale bar = 50μm).Data are presented as mean ± standard deviation from three independent experiments. Statistical significance was analyzed by two-tailed Student’s t-tests. ns = not significant, *P < 0.05, **P < 0.01, ***P < 0.001, ****P < 0.0001.

To examine the expression profile of WISP1 in fibroblasts, we successfully isolated CAFs and their paired NFs from tumor tissues and adjacent non-tumor tissues of ESCC samples. Immunofluorescence multiplex staining and WB analysis were employed to confirm the identity of CAFs and NFs based on their characteristic high expression of αSMA and FAP (**Figures 8A, B**). Furthermore, WB results demonstrated that WISP1 expression was significantly upregulated in CAFs compared with NFs (**Figure 8C**).

### 3.9 The influence of WISP1 expression on CAF functionality

To examine the impact of WISP1 on the functionality of CAFs, we utilized a lentiviral vector expressing shRNA to specifically knock down WISP1 expression. This knockdown was validated through qRT-PCR (P < 0.01) and WB analyses (**Figures 8D, E**).

Subsequent functional assays, including CCK8 (P < 0.001), EdU incorporation (P < 0.001), and Transwell migration/invasion experiments (P < 0.001), demonstrated that the depletion of WISP1 significantly attenuated the proliferation, migration, and invasive capabilities of CAFs compared to those in the empty vector control group (**Figures 8F–K**). To evaluate the knockdown efficiency of WISP1 at the secretory level, we performed an ELISA to quantitatively analyze WISP1 levels in the culture supernatants of untransfected CAFs, CAFs-shVector control group, and CAFs-shWISP1 cells. The results demonstrated that, at 0 h, 24 h, and 48 h time points, the levels of secreted WISP1 in CAFs-shWISP1 were significantly lower compared to those in both untransfected CAFs (P < 0.0001) and the CAFs-shVector control group (P < 0.0001). No significant difference was observed between untransfected CAFs and CAFs-shVector, indicating that lentiviral transduction alone does not affect WISP1 secretion (**Figure 8M**).

These findings, together with our previous data and existing literature, support the notion that CAFs play a pivotal role in ECM remodeling within the TME (18, 19).Based on these observations, we further assessed the expression levels of COL1A1 (P < 0.05) and MMP14 (P < 0.001) in CAFs following WISP1 knockdown via WB analysis. The results revealed a marked reduction in both COL1A1 and MMP14 expression in the WISP1-knockdown group relative to the empty vector control (**Figures 8L, O**). Notably, treatment with recombinant human WISP1 (rhWISP1) effectively restored the expression of COL1A1 (P < 0.01) and MMP14 (P < 0.01) in WISP1-depleted CAFs (**Figures 8L, O**).

### 3.10 The impact of WISP1 knockdown in CAFs on the function of ESCC cells

During tumorigenesis and progression, CAFs modulate the molecular composition of the ECM by enhancing collagen deposition and MMP expression, thereby remodeling the ECM to facilitate cancer invasion and metastasis (20–22).

To elucidate the effects of WISP1 knockdown in CAFs on the functional behavior of ESCC cells, we conducted indirect co-culture experiments using Transwell chambers (with or without Matrigel) for 48 hours, co-culturing KYSE150 and Eca109 cells with CAFs (**Figure 8N**). The results of the Transwell assays revealed that co-culture with CAFs-shWISP1 significantly attenuated the migratory and invasive capacities of both KYSE150 and Eca109 cells (P < 0.0001). Lentiviral transduction (shVector vs. Untransfected) does not alter CAFs’ ability to promote ESCC progression. The observed suppression of metastasis-related phenotypes is specifically attributable to WISP1 knockdown (**Figures 8O, Q**). To determine the optimal rescue concentration, we established a dose gradient (0, 0.2, 0.4, 0.6, 0.8 μg/mL) based on previous studies characterizing WISP1 bioactivity (23). Notably, rhWISP1 treatment led to a concentration-dependent increase in Snail expression in both KYSE150 and Eca109 cells, with the maximum effect observed at 0.8 μg/mL (**Supplementary Figures S3A, B**). This dose-response relationship informed our selection of 0.8 μg/mL for subsequent rescue experiments. Consistent with these molecular findings, supplementation of the CAFs-shWISP1 co-culture system with rhWISP1 (0.8 μg/mL) significantly restored the migratory and invasive capacities of KYSE150 and Eca109 cells (P < 0.0001) (**Figures 8R, S**).

### 3.11 WISP1 regulates ECM remodeling in CAFs via STAT3 signaling

**Figure 9:**
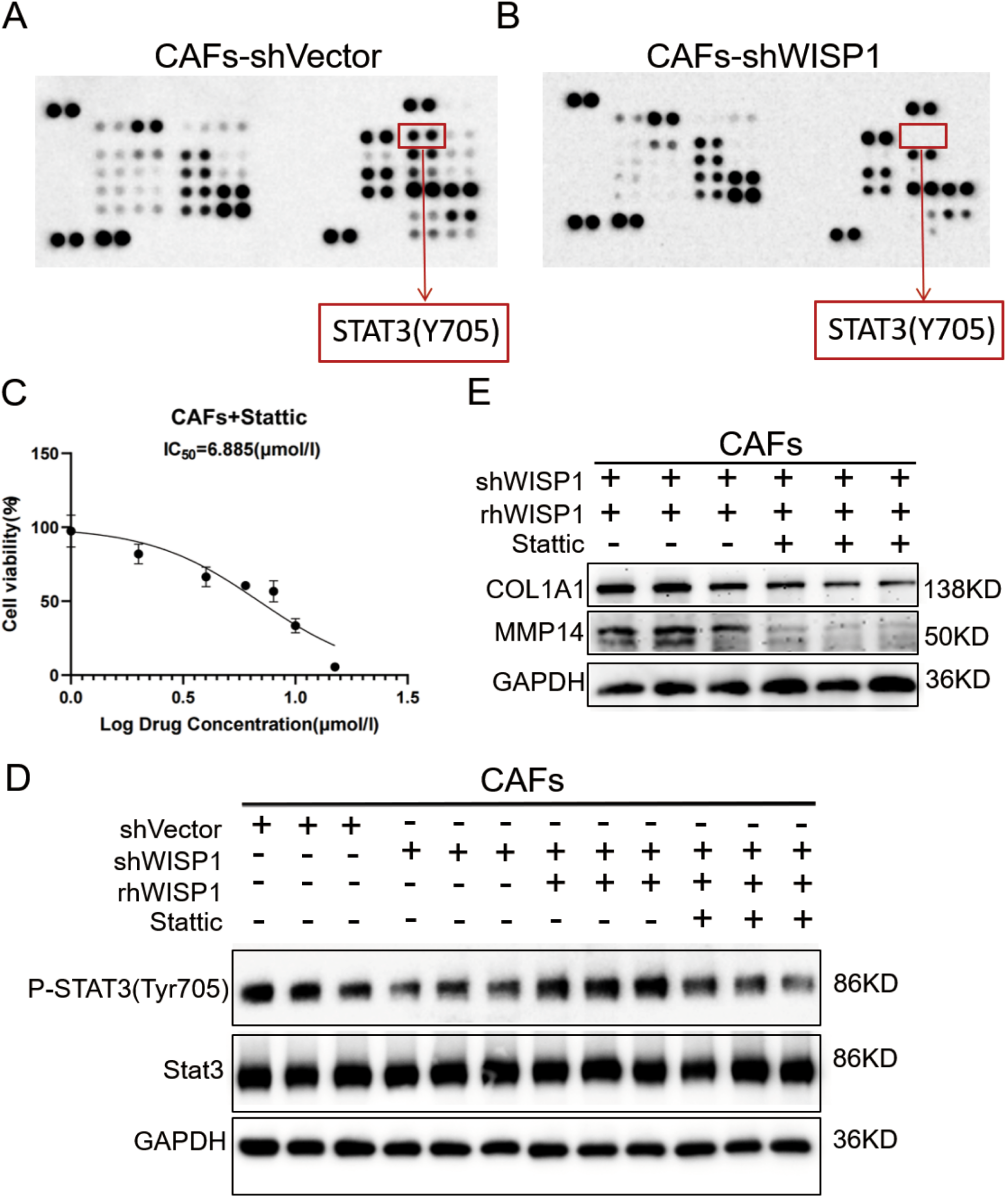
WISP1 regulates ECM remodeling in CAFs through STAT3 signaling. **(A, B)** Representative phospho-kinase antibody array membrane comparing CAFs-shVector and CAFs-shWISP1. Red boxes highlight phosphorylated STAT3 (Y705) signals. **(C)** Dose-response curve of STAT3 inhibitor Stattic in CAFs. **(D)** Western blot analysis of phosphorylated STAT3 (Y705), total STAT3, and GAPDH (loading control) in: CAFs-shVector, CAFs-shWISP1, CAFs-shWISP1 + rhWISP1 (0.8 μg/mL), and CAFs-shWISP1 + rhWISP1 (0.8 μg/mL) + Stattic (7 μM). **(E)** Western blot analysis of COL1A1, MMP14, and GAPDH in CAFs-shWISP1 + rhWISP1 (0.8 μg/mL) and CAFs-shWISP1 + rhWISP1 (0.8 μg/mL) + Stattic (7 μM).

To elucidate the downstream signaling mechanisms by which WISP1 regulates CAF function and ECM remodeling, we performed a phospho-kinase antibody array comparing CAFs-shVector and CAFs-shWISP1. This screening revealed a pronounced decrease in the phosphorylation of STAT3 at tyrosine 705 (STAT3 Y705) in WISP1-knockdown CAFs (**Figures 9A,B**). WB analysis confirmed this finding, showing significantly reduced p-STAT3 Y705 levels in CAFs-shWISP1 compared to control CAFs-shVector, without affecting total STAT3 protein expression (**Figure 9D**). Treatment of CAFs-shWISP1 with recombinant human WISP1 (rhWISP1, 0.8 µg/mL) effectively restored p-STAT3 Y705 levels (**Figure 9D**). Conversely, co-treatment with the STAT3 inhibitor Stattic (7 µM, determined as the IC50 value in CAFs, **Figure 9C**) abrogated the rhWISP1-induced phosphorylation of STAT3 (**Figure 9D**).

Building upon our previous observation that WISP1 knockdown suppresses COL1A1 and MMP14 expression (**Figures 8L,P**), we investigated the role of STAT3 in this regulation. While rhWISP1 treatment successfully restored COL1A1 and MMP14 expression in CAFs-shWISP1 (**Figures 8L,P**), the concurrent inhibition of STAT3 activation by Stattic blocked this restorative effect (**Figure 9E**). Collectively, these data establish that WISP1 modulates the expression of key ECM components COL1A1 and MMP14 in CAFs primarily through activation of the STAT3 signaling pathway.

## 4 Discussion

Analysis using the TIMER and GEPIA databases reveals that WISP1 exhibits differential expression across various cancer types, including EC. This finding underscores a significant association between WISP1 gene expression levels and diverse cancers, offering novel insights into the molecular mechanisms underlying carcinogenesis. Furthermore, our integrated analysis of four ESCC datasets, coupled with IHC and WB validation, demonstrates that WISP1 is markedly upregulated in ESCC and strongly correlates with poorer patient prognosis. Collectively, these results provide compelling evidence that WISP1 may play a critical role in the development and progression of ESCC.

Subsequently, through an integrative analysis of multiple datasets, we identified a significant association between WISP1 function and the organization and structure of the ECM. This finding is highly consistent with the biological characteristics of ESCC and offers novel insights into the role of WISP1 in this context. Moreover, the results of KEGG pathway analysis further corroborate the involvement of WISP1 in ESCC. Notably, the “ECM-receptor interaction” pathway, one of the key pathways implicated in this process, is closely linked to WISP1 function. This pathway plays a pivotal role in regulating cellular interactions with the ECM, and its dysregulation may lead to alterations in cell adhesion, migration, and invasion, thereby contributing to tumor initiation and progression (24).

Consequently, WISP1 may influence the progression of ESCC by participating in this pathway. In summary, this study demonstrates a robust functional relevance of WISP1 to ESCC through transcriptomic data analysis. As an ECM-associated gene, WISP1 exhibits differential expression in ESCC, and its function is significantly linked to the organization and structural integrity of the ECM. These findings not only deepen our understanding of the pathogenesis of ESCC but also provide a theoretical foundation for developing therapeutic strategies targeting WISP1.

Numerous existing studies have indicated that CAFs are closely associated with the prognosis of ESCC patients. In surgically treated ESCC patients, CAFs positively correlate with increased tumor invasiveness, lymph node metastasis, advanced clinical staging, and poorer overall survival (18). Furthermore, CAFs can serve as a prognostic indicator for ESCC patients undergoing neoadjuvant therapy (25). A recent study employing scRNA-seq revealed a substantial presence of CAFs within the TME of advanced ESCC (25). This evidence suggests that the formation of CAFs may represent a critical event in the progression of ESCC.

Our study, leveraging scRNA-Seq analysis, not only corroborated these findings but also further elucidated the expression profile and potential functions of the WISP1 gene within the ESCC microenvironment. Specifically, we detected significant upregulation of WISP1 in CAFs, providing novel insights into its specific role in ESCC progression. By stratifying fibroblast populations based on WISP1 expression levels, we observed distinct clustering of WISP1-positive fibroblasts in ESCC tissue samples, which exhibited enhanced signaling activity. This finding implies that WISP1 expression may be linked to functional reprogramming of fibroblasts and their contributions to the TME. GSEA revealed activated gene signatures within the WISP1-positive cluster, which are strongly associated with the organization and function of the ECM as well as immune regulation. These results suggest that WISP1 may facilitate ESCC progression by modulating the ECM and immune microenvironment. Furthermore, WISP1-positive clusters showed strong enrichment for EMT-related signatures [primarily involving ECM remodeling effectors like collagen and MMPs, rather than canonical EMT markers in CAFs (26–28)], along with coordinated activation of G2M checkpoint regulators and E2F transcriptional targets, mechanistically linking WISP1 to CAF-mediated stromal remodeling and tumor-promoting functions (29, 30).

An integrated analysis of three independent transcriptomic cohorts demonstrated that elevated WISP1 expression consistently activated signaling pathways associated with EMT. These findings collectively support the potential role of WISP1 in promoting tumor invasion and metastatic dissemination. Notably, while WISP1 overexpression exhibited suppressive effects on proliferation-related pathways in specific datasets, this observation is consistent with the well-established “Go-or-Grow” paradigm in cancer biology (31).

Mechanistically, the progression of EMT is generally characterized by a transient reduction in proliferative capacity, coinciding with an enhanced ability for migration and invasion (31). This inverse relationship implies that the WISP1-mediated inhibition of proliferation may constitute a critical component of its EMT-promoting function, thereby facilitating accelerated tumor progression via phenotypic switching.

Our experimental data indicate that the knockdown of WISP1 expression in CAFs significantly suppresses their proliferation, migration, and invasion capabilities. This finding directly underscores the pivotal role of WISP1 in regulating CAF functions. More importantly, we observed a marked downregulation in the expression of genes associated with ECM remodeling, such as COL1A1 and MMP14, upon WISP1 depletion. These results suggest that WISP1 may modulate the ECM remodeling capacity of CAFs by regulating the expression of these genes, thereby contributing to tumor progression. This discovery offers a novel mechanistic insight into how CAFs facilitate tumor invasion and migration, further linking the role of WISP1 to ECM remodeling processes. Additionally, our study elucidates the interaction between CAFs and ESCC cells and highlights its significance in tumor development. Through indirect co-culture experiments, we found that ESCC cells co-cultured with WISP1-knockdown CAFs exhibited reduced migratory and invasive abilities. Supplementation of rhWISP1 in CAFs-shWISP1 co-cultures effectively restored the migratory and invasive capacity of ESCC cells. This finding reinforces the essential role of WISP1 in mediating the tumor-promoting effects of CAFs and suggests that WISP1 may influence ESCC cell migration and invasion by modulating the ECM remodeling activity of CAFs. This association not only enhances our understanding of the mechanisms underlying the interaction between CAFs and ESCC cells but also identifies potential therapeutic targets for strategies aimed at inhibiting WISP1. There is compelling evidence demonstrating that CAFs can promote chemoresistance and immune escape in ESCC, underscoring their critical role in tumor progression and treatment resistance (18). Critically, our study elucidates a key molecular axis underlying WISP1’s pro-tumorigenic functions in CAFs. Beyond establishing WISP1’s role in driving CAF activation and ECM remodeling, we identified STAT3 phosphorylation at Tyr705 as a pivotal downstream signaling node. Phosphoproteomic screening and functional validation demonstrated that WISP1 knockdown suppresses STAT3 Y705 phosphorylation; recombinant WISP1 rescues this phosphorylation; and pharmacological STAT3 inhibition (Stattic) abrogates WISP1-mediated induction of COL1A1 and MMP14. This mechanistic cascade directly links WISP1 signaling to the STAT3-driven transcriptional regulation of ECM remodeling effectors—a finding that addresses the knowledge gap regarding how CAFs execute WISP1-dependent stromal reprogramming in ESCC.

In our study, the tumor microenvironment in the high WISP1 expression group was characterized by an unfavorable state, marked by reduced immune cell infiltration and decreased immune scores, stromal scores, and ESTIMATE scores. These findings suggest that high WISP1 expression may contribute to immune suppression, thereby facilitating tumor immune escape. Further analysis revealed upregulation of immune checkpoint inhibitory molecules such as PD-L1, CD276, and TIGIT in the high-expression group. These molecules play a critical role in tumor immune evasion. Notably, WISP1 expression exhibited a positive correlation with these molecules, indicating that WISP1 may be involved in their regulation. Although WISP1 is traditionally associated with ECM formation, the reduced stromal scores observed in the high-expression group appear to contradict this conventional understanding. Our research highlights that WISP1 plays a pivotal role in regulating CAF function. Compared to NFs, CAFs inherently possess a greater capacity for secreting ECM proteins. Our experiments further confirmed that WISP1 regulates two key ECM components and related enzymes: COL1A1 and MMP14. COL1A1 serves as one of the primary structural proteins in the ECM, while MMP14 mediates the degradation of multiple ECM components. The regulation of these factors by WISP1 through STAT3 signaling suggests that the ECM in the ESCC microenvironment exists in a dynamic state, where concurrent upregulation of structural (COL1A1) and degradative (MMP14) components drives rapid ECM turnover. The observed reduction in stromal scores likely reflects accelerated catabolism-counterbalanced ECM remodeling rather than diminished matrix production. Additionally, we found that WISP1 expression correlates with sensitivity to multiple drugs, implying that WISP1 may modulate tumor response to therapy. This finding provides valuable insights for developing intervention strategies targeting WISP1, which could enhance the efficacy of cancer treatment and address drug resistance.

In summary, the findings of this study enable us to construct a comprehensive and logically coherent model of action. The high expression of WISP1 in CAFs directly modulates their biological behaviors via STAT3 activation, encompassing proliferation, migration, invasion, and STAT3-dependent secretion of ECM components (COL1A1/MMP14). These altered CAFs subsequently influence ESCC progression through multiple mechanisms, such as promoting tumor cell migration and invasion by remodeling the ECM, inducing drug resistance via activation of resistance-related signaling pathways, and assisting tumor cells in evading immune surveillance by modulating the immune microenvironment. Thus, the WISP1-CAFs axis constitutes a critical regulatory component during ESCC development, offering a novel perspective for understanding its pathogenesis. Despite the significant advancements made in this study, certain limitations remain. For example, while we have demonstrated that the WISP1-STAT3 axis governs ECM remodeling in CAFs, further investigation is required to elucidate upstream regulators of WISP1 in the TME, potential STAT3-independent pathways co-regulated by WISP1, and the temporal dynamics of this signaling cascade during ESCC progression. Moreover, this study predominantly focuses on cellular levels and *in vitro* experiments; future research should incorporate more *in vivo* studies to comprehensively validate the role of the WISP1-CAFs axis in ESCC animal models and explore potential therapeutic strategies based on this axis. Through the integration of multi-omics technologies and functional experiments, this study systematically delineates the pivotal role of WISP1 within the ESCC TME and its intimate association with CAFs. The identification of the WISP1-CAFs axis provides new targets and directions for ESCC research, laying the groundwork for the development of more effective diagnostic tools and therapeutic strategies.

## Data Availability

The original contributions presented in the study are included in the article/**Supplementary Material**. Further inquiries can be directed to the corresponding authors.

## Supplementary material

Supplementary Figure 1 Tumor microenvironment and immune cell infiltration analysis in the GSE53624 dataset. **(A)** Differences in the abundance of infiltrating immune cells between high-expression and low-expression groups. **(B–D)** Differences in stromal score, immune score, and estimate score between high-expression and low-expression groups. **(E)** Evaluation of the expression of immune checkpoint molecules (CD274, PDCD1, TIGIT, CD276, CTLA4, LAG3) between high-risk and low-risk groups. **(F)** Correlation between WISP1 expression and immune checkpoint molecules (CD274, PDCD1, TIGIT, CD276, CTLA4, LAG3).Blue indicates positive correlation, red indicates negative correlation, and the numbers inside the boxes represent the magnitude of the correlation. *p < 0.05; **p < 0.01; ***p < 0.001; ****P < 0.0001.

Supplementary Figure 2 Depicts the cellular microenvironment of ESCC and normal esophageal tissues, revealing high expression of WISP1 in ESCC fibroblasts. **(A)** UMAP plot of all 22 clusters at Seurat resolution 3.0 which were used for the assignment of major cell types based on well-defined markers. **(B, C)** UMAP plots were generated at Seurat resolution 3.0 for clustering; global annotation of cell types was performed using specific markers. **(D)** The data were split into normal and tumor tissue groups for differential expression analysis. **(E)** Expression patterns of WISP1 and CAF-specific markers were analyzed across various cell types. **(F)** Differential expression between high-WISP1 and low-WISP1 cell clusters is presented.

Supplementary Figure 3 Western blot (WB) analysis of rhWISP1 dose-dependent effects on Snail expression. **(A, B)** WB analysis of Snail protein expression in KYSE150 and Eca109 cells treated with increasing concentrations of rhWISP1 (0–0.8 μg/mL) for 48 hours.
