# Supplementary figures and images for: WISP1 drives esophageal squamous cell carcinoma progression via modulation of cancer-associated fibroblasts and immune microenvironment
Source: Front Immunol. 2025 Jul 23;16:1586790. doi: 10.3389/fimmu.2025.1586790 (PMC12325208; doi:10.3389/fimmu.2025.1586790)

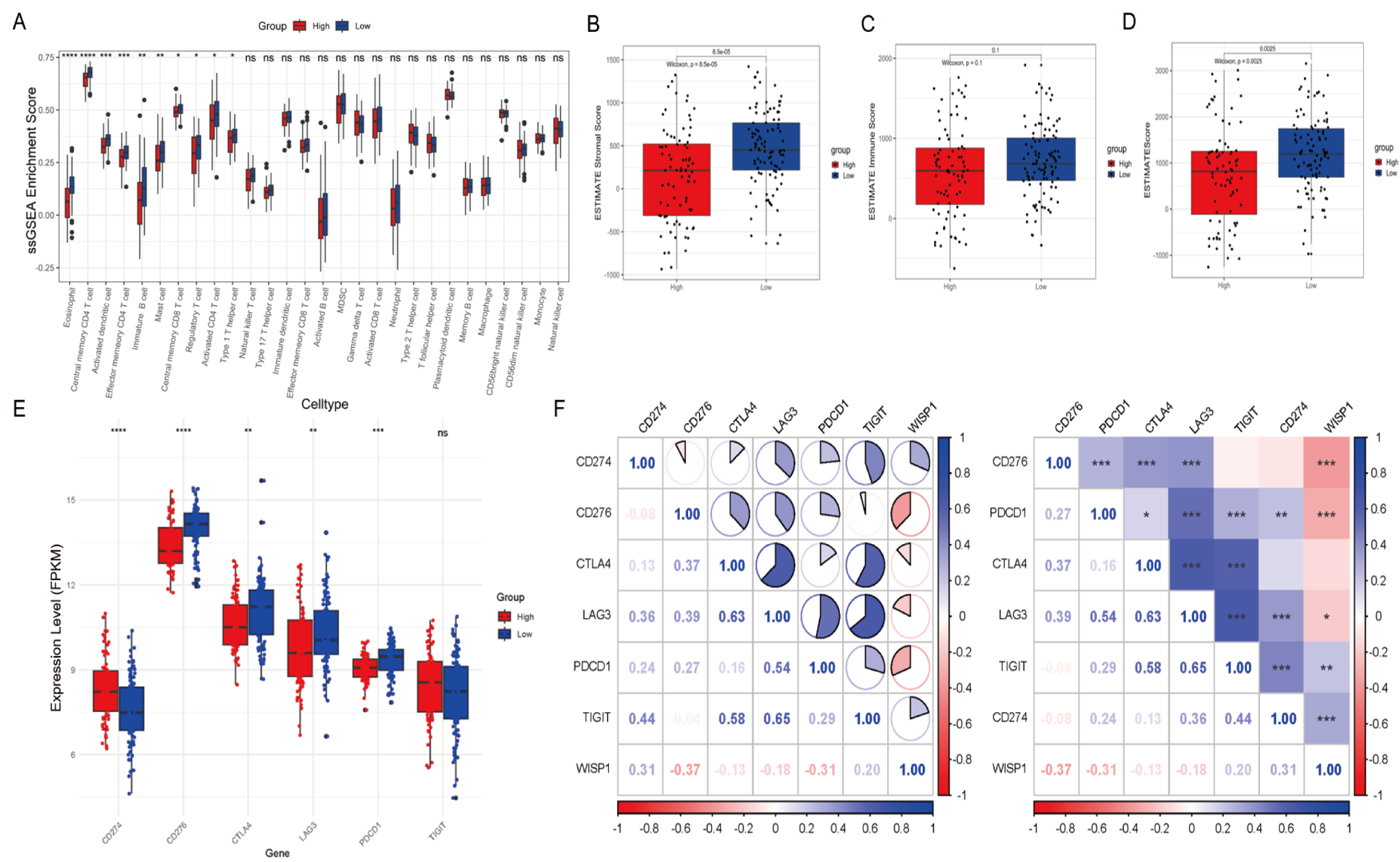

Supplement: Supplementary Figure 1 — Tumor microenvironment and immune cell infiltration analysis in the GSE53624 dataset. (A) Differences in the abundance of infiltrating immune cells between high-expression and low-expression groups. (B–D) Differences in stromal score, immune score, and estimate score between high-expression and low-expression groups. (E) Evaluation of the expression of immune checkpoint molecules (CD274, PDCD1, TIGIT, CD276, CTLA4, LAG3) between high-risk and low-risk groups. (F) Correlation between WISP1 expression and immune checkpoint molecules (CD274, PDCD1, TIGIT, CD276, CTLA4, LAG3).Blue indicates positive correlation, red indicates negative correlation, and the numbers inside the boxes represent the magnitude of the correlation. *p < 0.05; **p < 0.01; ***p < 0.001; ****P < 0.0001. [file Image1.tif]

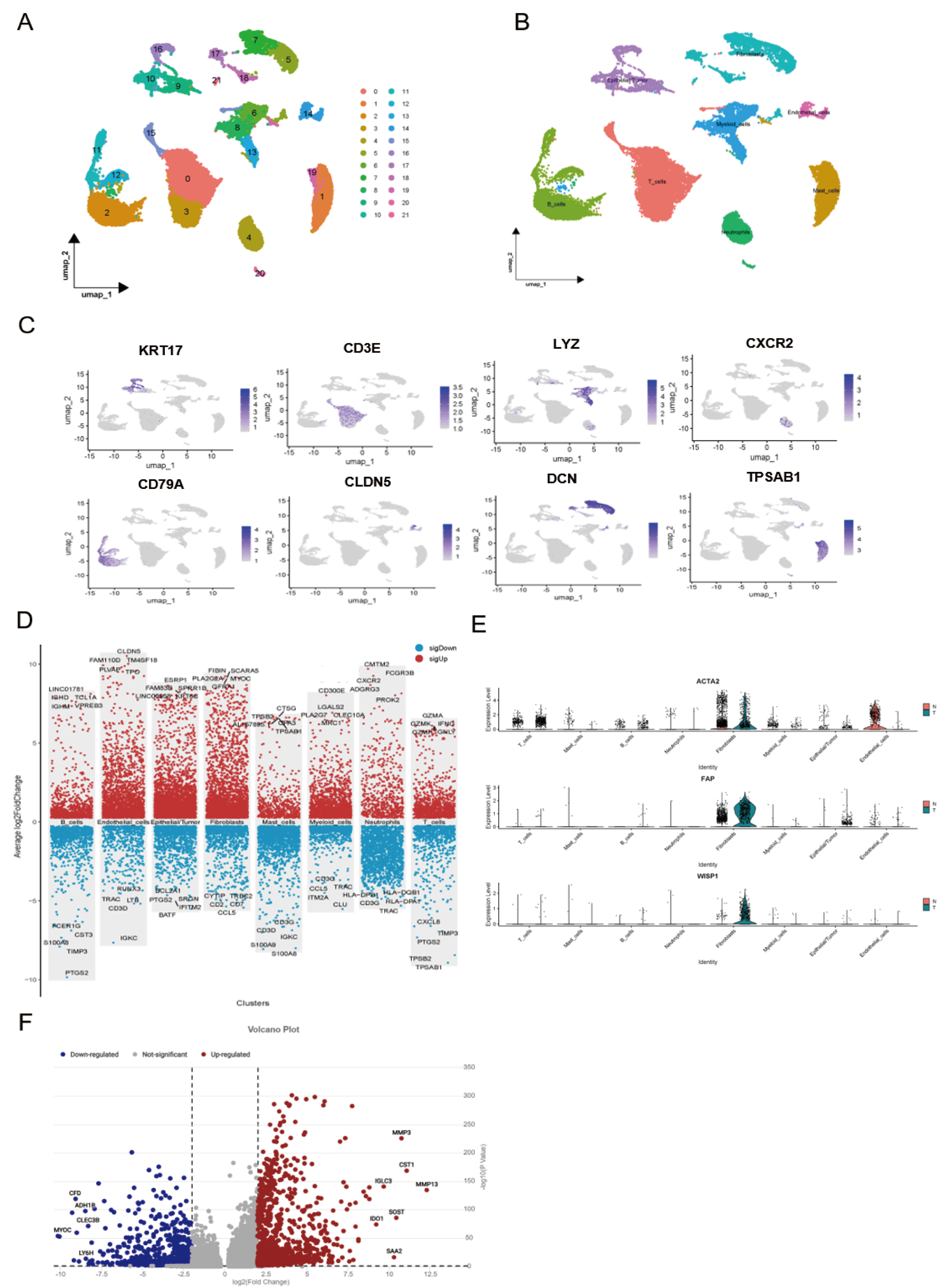

Supplement: Supplementary Figure 2 — Depicts the cellular microenvironment of ESCC and normal esophageal tissues, revealing high expression of WISP1 in ESCC fibroblasts. (A) UMAP plot of all 22 clusters at Seurat resolution 3.0 which were used for the assignment of major cell types based on well-defined markers. (B, C) UMAP plots were generated at Seurat resolution 3.0 for clustering; global annotation of cell types was performed using specific markers. (D) The data were split into normal and tumor tissue groups for differential expression analysis. (E) Expression patterns of WISP1 and CAF-specific markers were analyzed across various cell types. (F) Differential expression between high-WISP1 and low-WISP1 cell clusters is presented. [file Image2.tif]

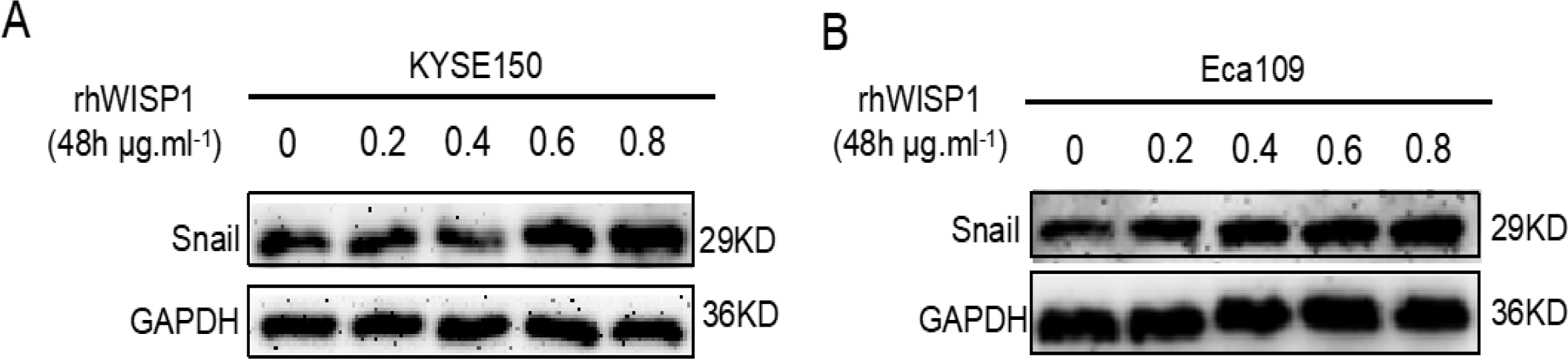

Supplement: Supplementary Figure 3 — Western blot (WB) analysis of rhWISP1 dose-dependent effects on Snail expression. (A, B) WB analysis of Snail protein expression in KYSE150 and Eca109 cells treated with increasing concentrations of rhWISP1 (0–0.8 μg/mL) for 48 hours. [file Image3.tif]

Figure 8C

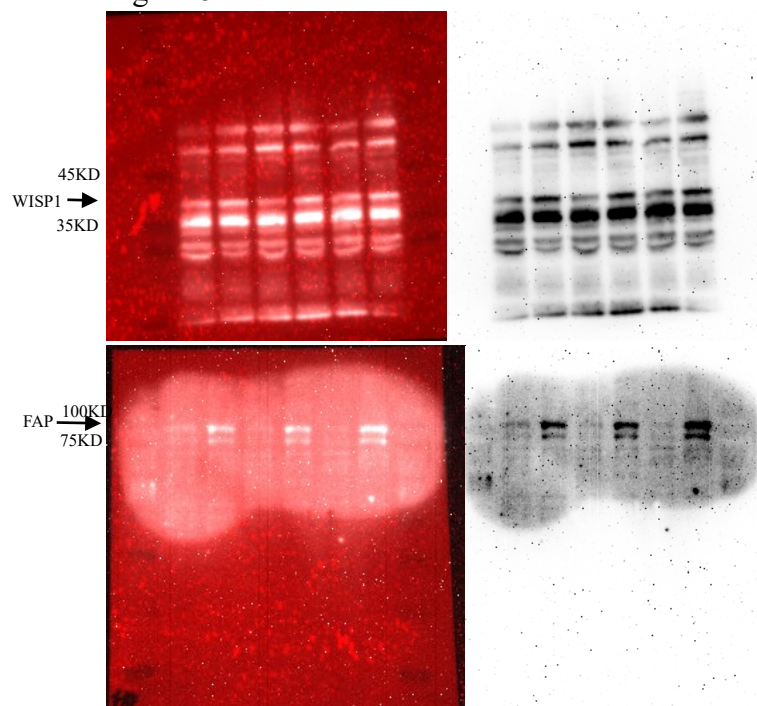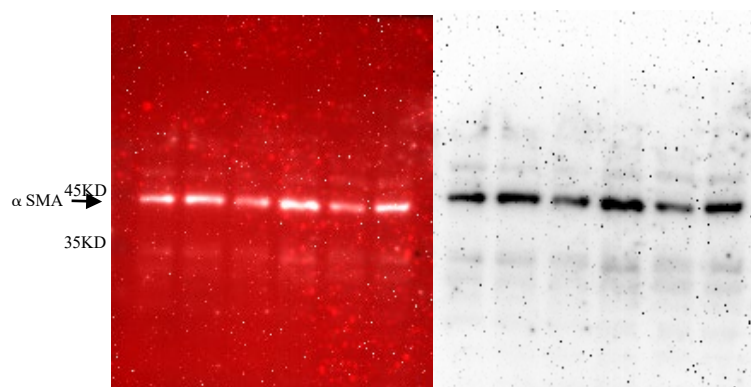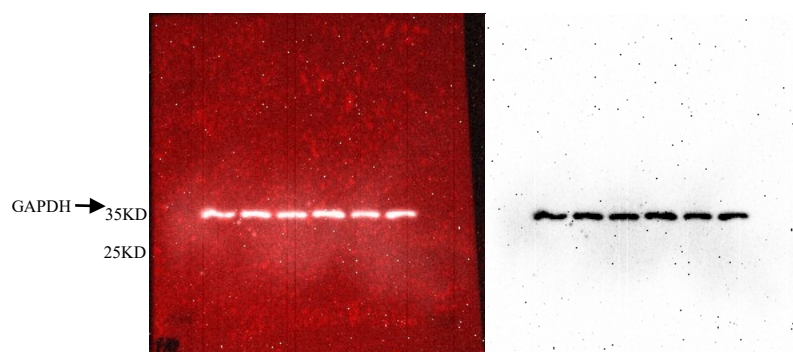

Figure 8E

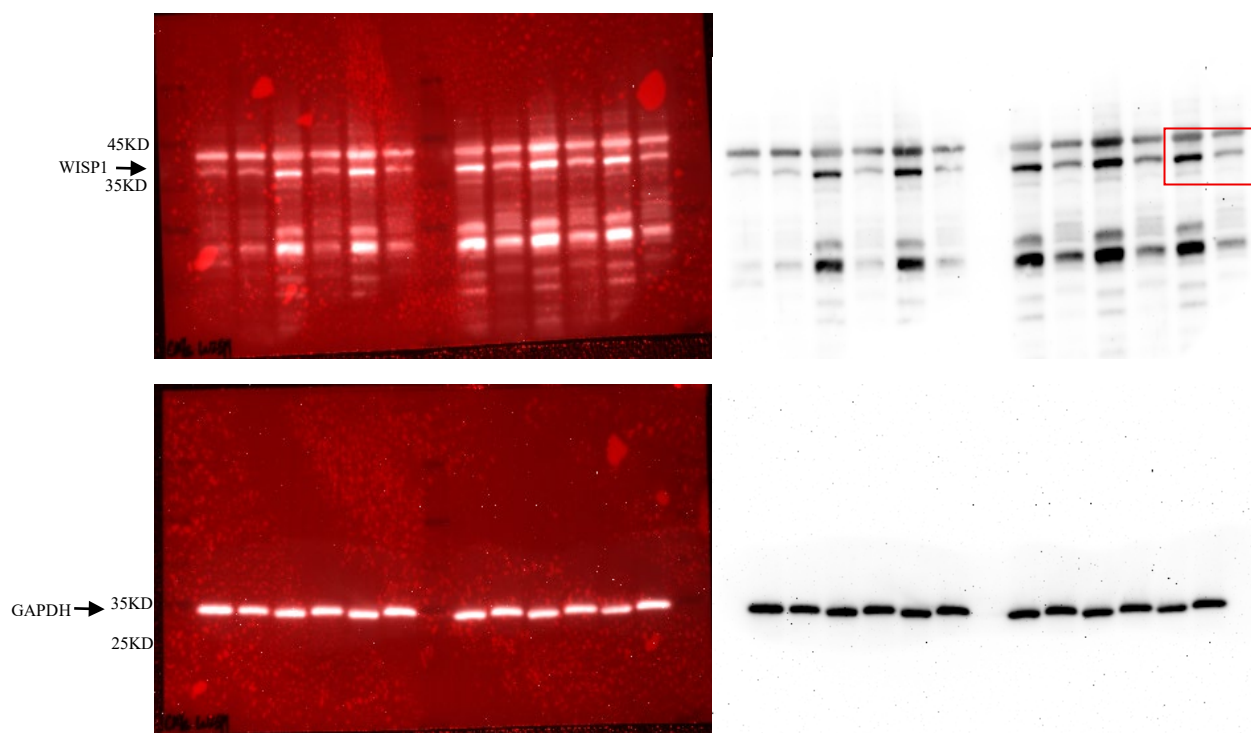

Figure 8L

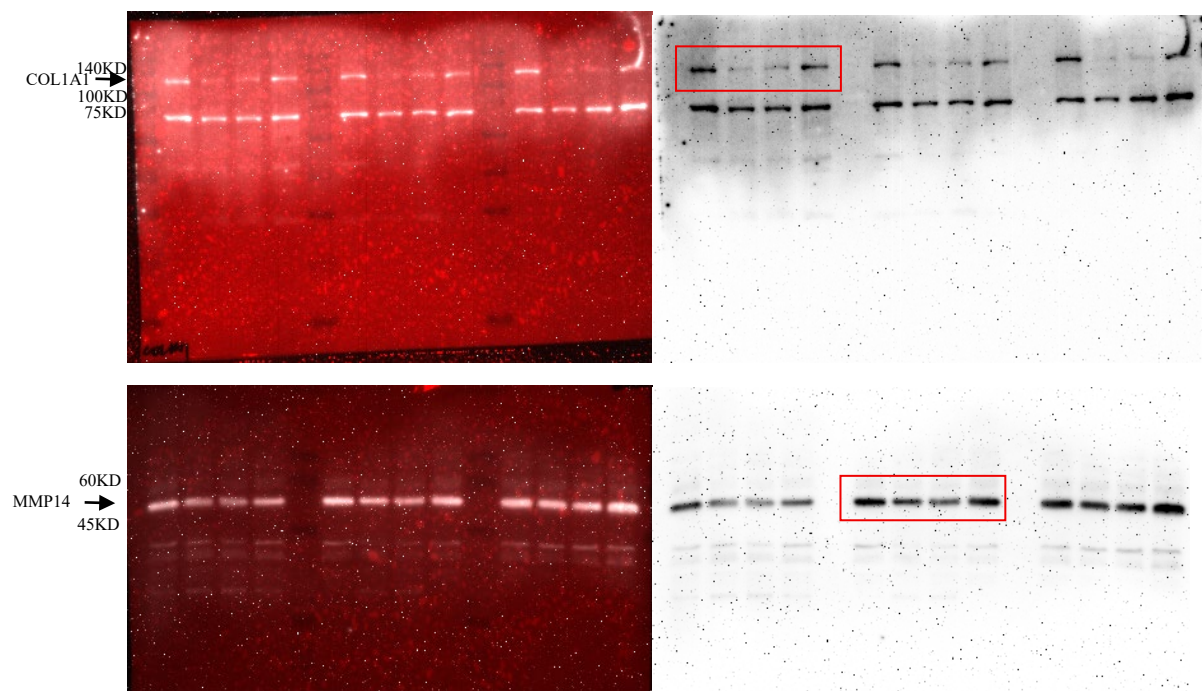

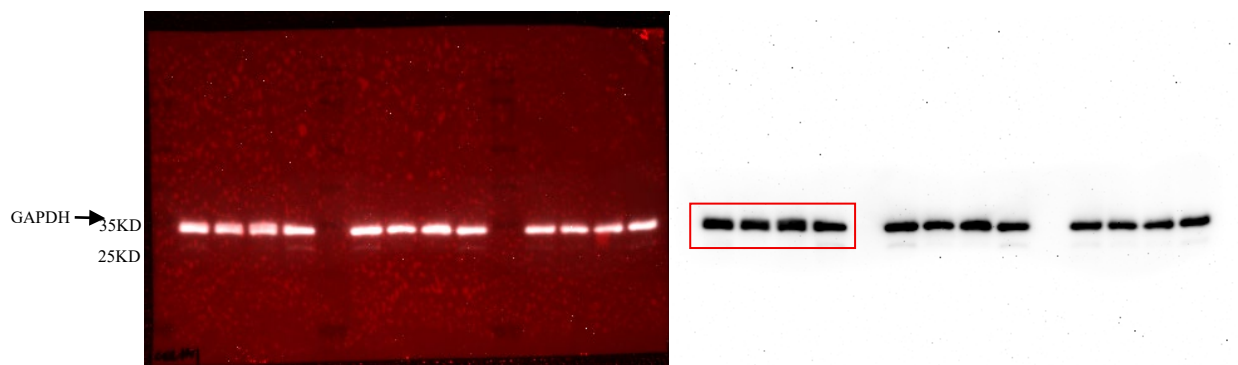

Figure 9

CAFs-shVector

CAFs-shWISP1

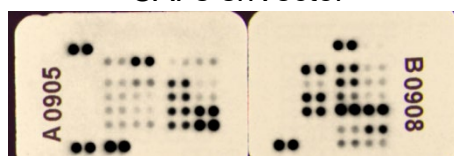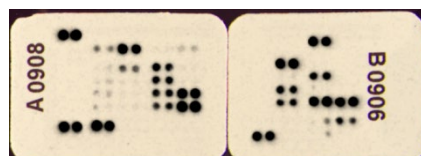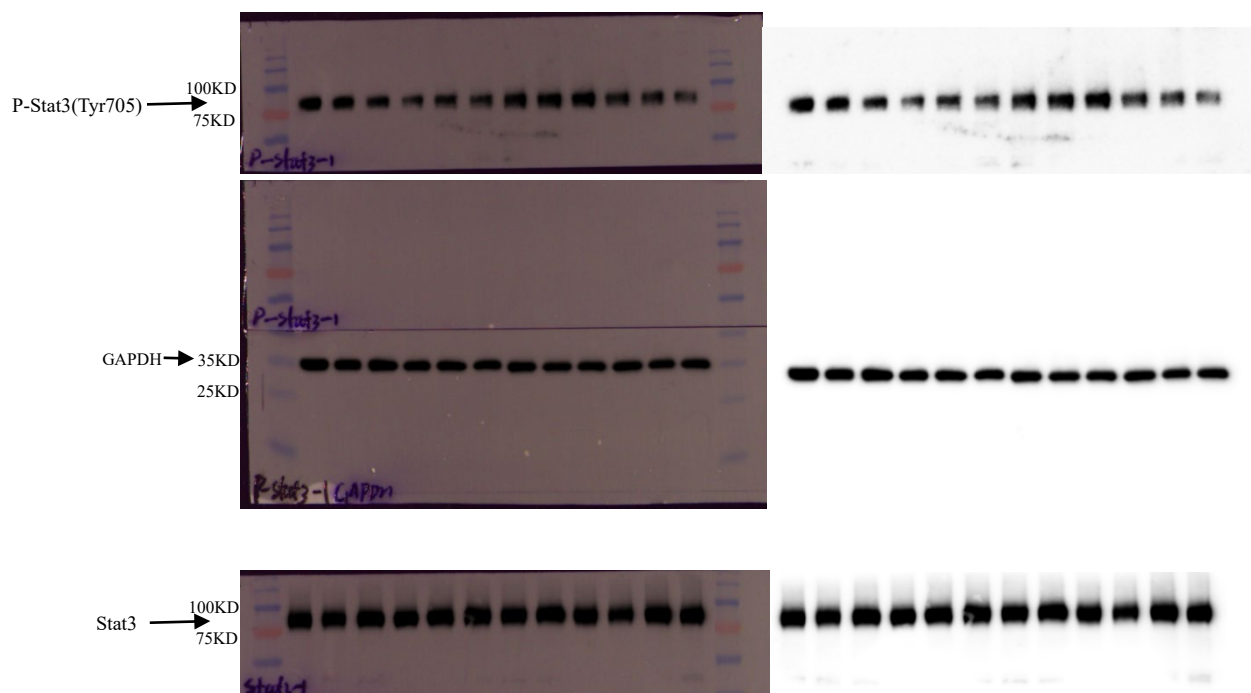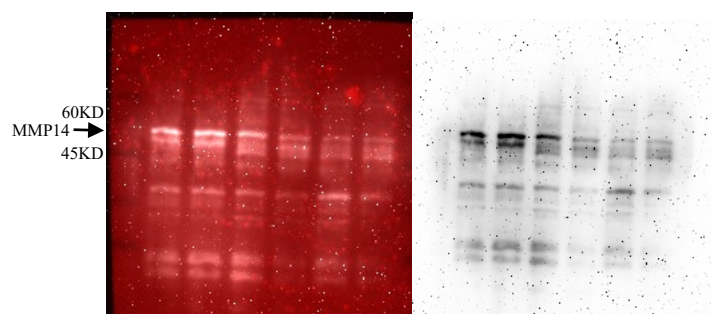

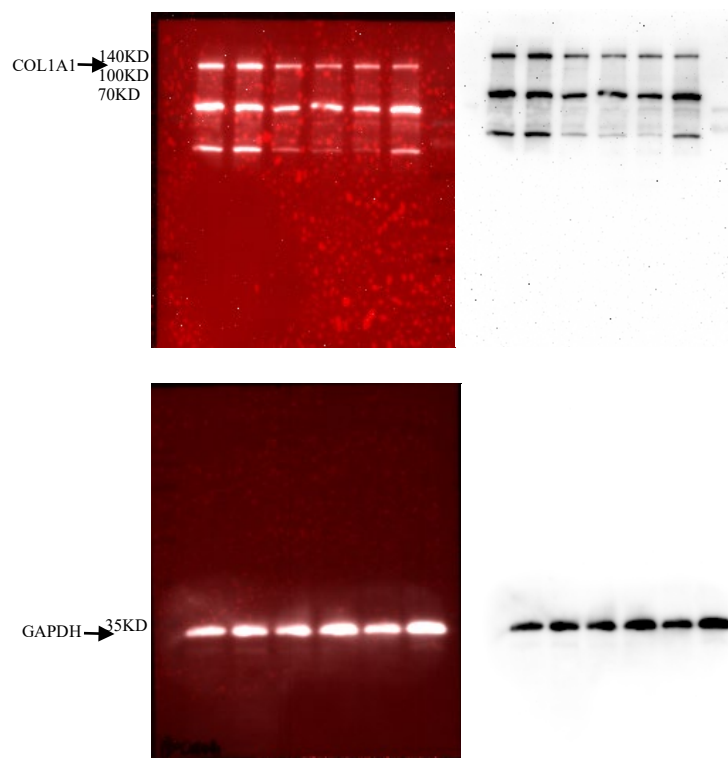

Figure S3  
KYSE150

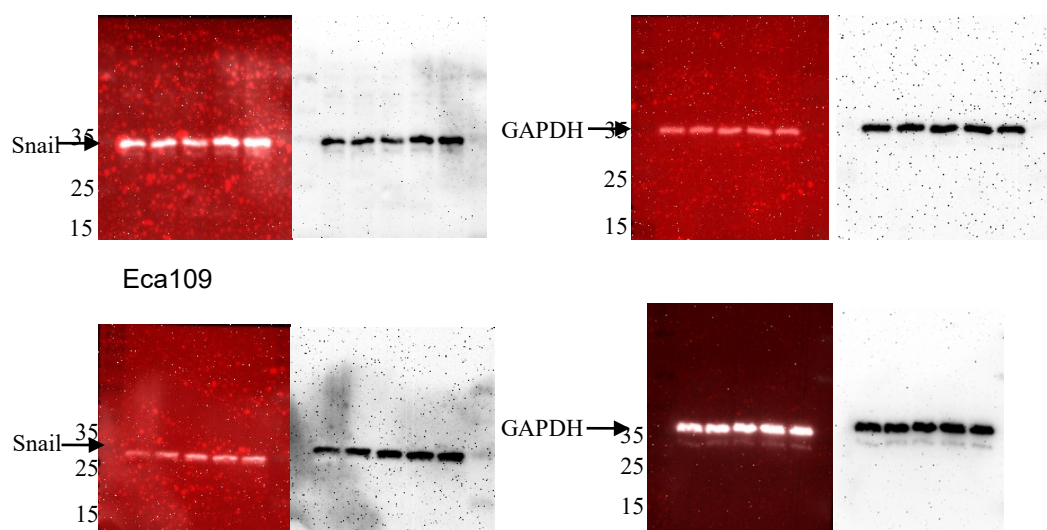

Supplement: Supplementary file 13 [file DataSheet9.pdf]
