# Supplementary material for: WISP1 drives esophageal squamous cell carcinoma progression via modulation of cancer-associated fibroblasts and immune microenvironment
Source: Front Immunol. 2025 Jul 23;16:1586790. doi: 10.3389/fimmu.2025.1586790 (PMC12325208; doi:10.3389/fimmu.2025.1586790)
Supplement: Supplementary file 4 [file Table1.docx]

Table S1

**Clinical features of the ESCC patients from GEO**

| **Features** | **GSE161533** | **GSE53624** | **GSE53625** | **TCGA** |
| --- | --- | --- | --- | --- |
| Age (years) |  |  |  |  |
| ≤60 | 8 | 61 | 90 | 54 |
| >60 | 20 | 58 | 89 | 28 |
| Sex |  |  |  |  |
| Female | 7 | 21 | 33 | 13 |
| Male | 21 | 98 | 146 | 69 |
| T stage |  |  |  |  |
| x |  |  |  | 2 |
| 1 |  | 8 | 12 | 8 |
| 2 |  | 20 | 27 | 29 |
| 3 |  | 62 | 110 | 39 |
| 4 |  | 29 | 30 | 4 |
| N stage |  |  |  |  |
| x |  |  |  | 3 |
| 0 |  | 54 | 83 | 50 |
| 1 |  | 42 | 62 | 24 |
| 2 |  | 13 | 22 | 2 |
| 3 |  | 10 | 12 | 3 |
| TNM stage |  |  |  |  |
| x |  |  |  | 2 |
| 1 | 9 | 6 | 10 | 5 |
| 2 | 11 | 47 | 77 | 52 |
| 3 | 8 | 66 | 92 | 19 |
| 4 |  |  |  | 4 |
| Survival status |  |  |  |  |
| Alive |  | 46 | 76 | 57 |
| Dead |  | 73 | 103 | 25 |

Table S2

**Primer sequences used in the study**

| Primer name | Primer sequences |
| --- | --- |
| WISP1/CCN4 sense: | 5’-AAGGTCAGCCCGTTTCAGAAGG-3’ |
| WISP1/CCN4 antisense: | 5’-AGCAGGACAAGGGAGAAGATTCAAG-3’ |
| GAPDH sense: | 5’-CCAGCAAGAGCACAAGAGGAAGAG-3’ |
| GAPDH antisense: | 5’-GGTCTACATGGCAACTGTGAGGAG-3’ |

Table S3

**The primary antibodies used are listed below**

| Antibodies | Source | Dilution ratio |
| --- | --- | --- |
| WISP1 | Proteintech, 18166-1-AP | 1:1000 |
| αSMA | Proteintech, 14395-1-AP | WB(1:1000),IF(1:100) |
| FAP | Proteintech, 11779-1-AP | WB(1:1000),IF(1:100) |
| MMP14 | Proteintech, 14552-1-AP | 1:1000 |
| COL1A1 | Proteintech, 67288-1-Ig | 1:5000 |
| Snail | Proteintech, 13099-1-AP | 1:1000 |
| GAPDH | Proteintech, 10494-1-AP | 1:5000 |
| HRP-conjugated Goat Anti-Rabbit IgG(H+L) | Proteintech, SA00001-2 | 1:10000 |
